# Single Stranded Fully Modified-Phosphorothioate Oligonucleotides can Induce Structured Nuclear Inclusions, Alter Nuclear Protein Localization and Disturb the Transcriptome *In Vitro*

**DOI:** 10.3389/fgene.2022.791416

**Published:** 2022-04-06

**Authors:** Loren L. Flynn, Ruohan Li, Ianthe L. Pitout, May T. Aung-Htut, Leon M. Larcher, Jack A. L. Cooper, Kane L. Greer, Alysia Hubbard, Lisa Griffiths, Charles S. Bond, Steve D. Wilton, Archa H. Fox, Sue Fletcher

**Affiliations:** ^1^ Centre for Molecular Medicine and Innovative Therapeutics, Health Futures Institute, Murdoch University, Murdoch, WA, Australia; ^2^ Perron Institute, Nedlands, WA, Australia; ^3^ Centre for Neuromuscular and Neurological Disorders, The University of Western Australia, Nedlands, WA, Australia; ^4^ Black Swan Pharmaceuticals, Wake Forest, NC, United States; ^5^ Cell and Tissue Therapies WA, Royal Perth Hospital, Perth, WA, Australia; ^6^ School of Human Sciences, The University of Western Australia, Nedlands, WA, Australia; ^7^ PYC Therapeutics, Nedlands, WA, Australia; ^8^ Centre for Microscopy, Characterization and Analysis, The University of Western Australia, Nedlands, WA, Australia; ^9^ Anatomical Pathology, Department of Health, Nedlands, WA, Australia; ^10^ School of Molecular Sciences, The University of Western Australia, Nedlands, WA, Australia

**Keywords:** antisense oligonucleotides, RNA analogue, RNA processing, paraspeckle nuclear organelles, phosphorothioate, morpholino oligomer, nuclear lamina

## Abstract

Oligonucleotides and nucleic acid analogues that alter gene expression are now showing therapeutic promise in human disease. Whilst the modification of synthetic nucleic acids to protect against nuclease degradation and to influence drug function is common practice, such modifications may also confer unexpected physicochemical and biological properties. Gapmer mixed-modified and DNA oligonucleotides on a phosphorothioate backbone can bind non-specifically to intracellular proteins to form a variety of toxic inclusions, driven by the phosphorothioate linkages, but also influenced by the oligonucleotide sequence. Recently, the non-antisense or other off-target effects of 2′ *O*- fully modified phosphorothioate linkage oligonucleotides are becoming better understood. Here, we report chemistry-specific effects of oligonucleotides composed of modified or unmodified bases, with phosphorothioate linkages, on subnuclear organelles and show altered distribution of nuclear proteins, the appearance of highly stable and strikingly structured nuclear inclusions, and disturbed RNA processing in primary human fibroblasts and other cultured cells. Phosphodiester, phosphorodiamidate morpholino oligomers, and annealed complimentary phosphorothioate oligomer duplexes elicited no such consequences. Disruption of subnuclear structures and proteins elicit severe phenotypic disturbances, revealed by transcriptomic analysis of transfected fibroblasts exhibiting such disruption. Our data add to the growing body of evidence of off-target effects of some phosphorothioate nucleic acid drugs in primary cells and suggest alternative approaches to mitigate these effects.

## Introduction

Antisense oligonucleotide (AO) drugs are a class of therapeutics designed to alter gene expression through binding RNA. ‘RNase H1 activating’ AOs contain modified chemical backbone nucleotides such as core DNA sequences flanked by modified ribonucleotide bases on a phosphorothioate backbone, and trigger RNase H1 mediated degradation of their target RNAs. Another class of AOs, comprised of homogenous modified nucleotide and backbone chemistry, instead acts by steric hinderance and alters conformation or prevents factors binding to the target RNA, thereby altering splicing, translation or RNA processing, rather than inducing degradation of the targeted transcript. Several of this latter class of AOs are now approved for use in diseases such as spinal muscular atrophy (SMA) (Paton, 2017) and a subset of Duchenne muscular dystrophy (DMD) (Mendell et al., 2013; Mendell et al., 2016). Within the steric inhibition class of therapeutics, it is not only the targeting sequence that distinguishes each AO, but also the backbone chemistry used. For example, *Nusinersen* (*Spinraza*) is a fully modified 2′ *O*-methoxyethyl AO on a phosphorothioate backbone, targeting a splice silencer (*ISS-N1*) in *SMN2* intron 7, that promotes exon 7 selection during pre-mRNA splicing (Singh et al., 2006). In contrast, *Exondys51, Vyondys53*, *Vitolarsen* and *Amondys45* are phosphorodiamidate morpholino oligomers (PMO) that target the *DMD* pre-mRNA to exclude exon 51, 53 (*Vyondys* and *Vitolarsen*) or 45, respectively, in order to re-frame the dystrophin transcript around frame shifting deletions flanking the respective exons, as a treatment for Duchenne muscular dystrophy (Mendell et al., 2013; Clemens et al., 2020; Frank et al., 2020; Shirley, 2021).

Strategies to improve biological stability and confer pharmaceutical properties to nucleic acid drugs include chemical modifications of the bases and nucleic acid backbone to increase resistance to endogenous nucleases; modifications that influence specific oligomer stability and activity, and conjugates that enhance cellular uptake and improve tissue distribution. While the phosphorothioate backbone is the most widely applied chemical modification, changes to the ribose moiety (eg, 2′ *O*-methylation, 2′ *O*-methoxyethyl, 2′ *O*-fluoro), nucleobases and other backbone modifications, including peptide nucleic acids and phosphorodiamidate morpholino oligomers, can confer specific characteristics and mechanisms of action [for review see (Veedu, 2015; Wilton et al., 2015; Le et al., 2016)]. Phosphorothioate backbone modification of nucleic acid drugs can engender non-specific interactions with endogenous cellular components. Early studies indicated that negatively charged phosphorothioate AOs bind to heparin-like proteins, laminin and collagen (Dias and Stein, 2002).

Over the last decade a significant body of work has revealed that ‘gapmer’ phosphorothioate AOs interact with many intracellular proteins (Crooke et al., 2020), providing an explanation for the observed AO toxicity. One class of proteins that interacts with phosphorothioate-containing AOs are nuclear paraspeckle-associated proteins of the drosophila behaviour/human splicing (DBHS) family, including SFPQ, NONO (also known as p54nrb) and PSPC1. Paraspeckles and paraspeckle proteins are involved in transcriptional regulation, transport and splicing pathways and their perturbation has consequences for cell and organism physiology and disease (Bond and Fox, 2009; Naganuma et al., 2012). The gapmer phosphorothioate AO interactions result in sequestration and mis-localisation of these proteins away from paraspeckles (Shen W. et al., 2014; Shen et al., 2015) and into a variety of other structures such as phosphorothioate AO-seeded nuclear and cytoplasmic foci, nuclear filamentous inclusions and to the nucleolus. Whilst the phosphorothioate backbone chemistry largely drives these off-target effects, sequence-specific effects are exhibited by some gapmer phosphorothioate AO sequences that are more prone to induce toxic effects than others. The ‘toxic’ gapmer AOs are suggested to induce cellular toxicity through nucleolar stress, mis-regulation of ribosomal RNA and caspase activation, as a consequence of the DNA component of the AO interacting with RNA recognition motifs in NONO, initiating RNase H1 recruitment into the complex (Shen et al., 2019; Vickers et al., 2019). A broader question relates to the global consequences on cellular processes arising from the interaction of AOs with nuclear proteins in many cell types. A further unresolved issue is if these effects are reproduced by fully modified phosphorothioate AOs that act through steric blocking, as opposed to the RNase H1-inducing gapmers used in other studies, since the mechanisms of action differ.

Investigating the consequences of non-specific phosphorothioate AO protein binding is particularly relevant when developing therapeutic antisense oligomer applications. Delivery of 2′ fluoro-modified gapmer phosphorothioate AOs to mice led to sequestration of paraspeckle proteins, in particular, DBHS proteins that was associated with acute hepatotoxicity, inflammation and apoptosis (Shen et al., 2018). While the 2018 study by Shen et al. (2018) focused on the severity of 2′ fluoro-modified phosphorothioate effects, all 2′ modifications evaluated sequestered paraspeckle proteins, albeit to varying degrees (Shen W. et al., 2014; Liang et al., 2015). Nevertheless, it has been possible to alter sequences to negate the undesirable effects of gapmers by limiting the number of phosphorothioate modified bases to include some bases on a phosphodiester backbone (Shen et al., 2018; Shen et al., 2019). Ottesen et al. (2021) reported massive perturbation of the transcriptome in SMA patient cells treated with 100 nM of phosphorothioate modified AO targeting *SMN2 ISS-N1*, that also triggered widespread aberrant splicing and altered expression of genes involved in transcription, splicing, translation, cell signaling, cell cycle, macromolecular trafficking, cytoskeletal dynamics, and innate immunity. The off-target effects were mitigated to some extent by using shorter *ISS-N1*-targeting AOs (Ottesen et al., 2021).

The backbone-dependent binding of phosphorothioate-modified oligonucleotides to platelets *in vitro* and *in vivo*, mediated by the platelet-specific receptor glycoprotein VI (GPVI) is also of note (Flierl et al., 2015), considering the broad range of nucleic acid therapeutics currently under investigation [for review see (Bennett et al., 2017)]. Oligonucleotides on a phosphorothioate backbone elicited strong platelet activation, effects on signaling, reactive oxygen species generation, adhesion, spreading, aggregation and thrombus formation (Flierl et al., 2015). Of particular relevance, 2′ *O*-methyl phosphorothioate AOs were reported to activate innate immunity when administered directly to the central nervous system in mice (Toonen et al., 2018). Recent data from the Phase 1/2 clinical trials of *Tofersen*, a gapmer designed to suppress SOD1 as a treatment for amyotrophic lateral sclerosis (ALS), reported pleocytosis within the CSF in 42% of the trial participants (Miller et al., 2020).

Here, we investigate fully 2′ *O*-methyl modified phosphorothioate AOs transfected into cells *in vitro* and show that they induce cellular inclusions and filaments that disrupt normal cell function. We focus on a relevant model of normal human cells, primary fibroblasts, and use 2′*O*- fully modified phosphorothioate backbone AOs, including standard control sequences provided by suppliers. Upon transfection of these AOs, we find intranuclear inclusions begin to form rapidly and appear stable once formed and may remain evident on the culture substrate, even after disintegration of the cell. Although the inclusions form most readily at high AO concentrations, these are detectable in fibroblasts transfected at concentrations as low as 12.5 nM. Notably, we show that these inclusions do not form upon transfection of annealed complementary, duplex phosphorothioate AOs, or duplex DNA:phosphorothioate oligomers, or the same sequences synthesised as the PMO chemistry. Super-resolution microscopy reveals the inclusions to be highly structured fibril-like aggregates that contain DBHS proteins along their length, with FUS at the termini. Striking transmission electron microscopy images of AO-transfected cells revealed regular nuclear structures, reminiscent of amyloid deposits, with internal longitudinal striations that are a regular distance apart, as well as electron dense regions at the termini that correspond to FUS-rich terminal regions seen in fluorescent images.

Others have shown that nucleoli are perturbed by gapmer phosphorothioate AO transfection and here we show that, in addition to nucleoli, Cajal bodies and splicing speckles are also perturbed, supporting nucleolar stress and mis-regulated ribosomal RNA processing as mechanisms for cellular toxicity. Finally, transcriptome analysis of 2′ modified phosphorothioate AO transfected fibroblasts revealed significant disruptions to chromatin biology, regulation of autophagy, nucleotide excision repair, membrane and organelle organization, apoptosis, signaling and protein transmembrane transport. Together our data suggests that, as with gapmers, 2′ fully modified phosphorothioate AOs cause damaging nuclear inclusions in normal human cells, disrupting RNA biology. Studies that use the phosphorothioate backbone chemistry may be confounded by non-antisense effects on many nuclear proteins and global disturbance to the transcriptome. We further report that the off-target effects can be somewhat mitigated by rendering phosphorothioate-containing oligonucleotides double-stranded.

## Materials and Methods

### Antisense Oligonucleotides

Phosphorodiamidate morpholino oligomers (PMOs) were purchased from Gene Tools LLC (Philomath, OR, United States), 2′ *O*-methyl phosphorothioate AOs were synthesised by TriLink BioTechnologies (San Diego CA, United States) and DNA phosphodiester AOs were synthesized by Integrated DNA Technologies (Singapore). The following sequences were evaluated; an AO encompassing the *SMN* intron 7 *ISS-N1* target, but with a longer sequence *SMN7D(-10-29)* (5′AUU​CAC​UUU​CAU​AAU​GCU​GG 3′); the Gene Tools standard control oligomer, only known to be biologically active in reticulocytes carrying a splice mutation in the human beta-globin pre-mRNA, as an unrelated sham control AO sequence (5′CCU​CUU​ACC​UCA​GUU​ACA​AUU​UAU​A 3′), and identified as ‘control AO’ throughout this study; *SMN7A(+13+32)* (5′CAC​CUU​CCU​UCU​UUU​UGA​UU 3′), designed to induce *SMN* exon 7 skipping (Flynn et al., 2021) and a *SMN* sense AO (5′ CCA​GCA​UUA​UGA​AAG​UGA​AU 3′) complementary to *SMN7D(-10-29).*

### Transfection

The 2′ *O*-methyl phosphorothioate AOs were transfected into dermal fibroblasts as lipoplexes using 3 µl of Lipofectamine 3000 (Life Technologies, Melbourne, Australia) per 1 ml of OptiMEM, according to the manufacturer’s protocol. The transfection mix was applied to cells seeded at 10,000 per cover slip for immunofluorescence and 15,000 cells per well in 24 well plates for RNA extraction and incubated (37°C) for 24 h prior to RNA and protein analysis. SMA patient fibroblasts were used for the experiments shown in Figures 8D–F (Coriell Cell Repositories, GM03813) and fibroblasts from our in-house biobank, obtained from a healthy volunteer, with informed consent (Murdoch University Human Research Ethics Committee Approvals #2013/156 and 2017/101) were used for all other experiments, except live-cell imaging (see below).

The PMOs were delivered to cells either uncomplexed (10 µM) and incubated for 72 h, annealed to a complementary sense DNA oligonucleotide (phosphodiester leash) and transfected as a lipoplex (100 nM) using Lipofectamine 3000 and incubated for 24 h, or by electroporation using a Neon Electroporation System (ThermoFisher Scientific, Melbourne, Australia) according to the manufacturer’s instructions. PMOs were delivered at 50 or 10 μM, using the following parameters: 1,650 V, 10 ms, 3 pulses, and the cells incubated for 24 h.

### RT-PCR on AO Transfected Cells

RNA was extracted using the MagMAX-96 Total RNA Isolation Kit, including DNase treatment (Life Technologies), according to the manufacturer’s instructions. RT-PCRs were performed using the One-step Superscript III RT-PCR kit with Platinum Taq polymerase (Life Technologies) according to the manufacturer’s instructions. All primer sequences and PCR conditions used in this study are detailed in Supplementary Table S1.

### cDNA Synthesis and Quantitative PCR

cDNA was synthesised from (∼500 ng) total RNA extracted from treated and untreated cell cultures using the Superscript IV first-strand synthesis system (Life Technologies) as per the manufacturer’s instructions. Prior to qPCR amplification, cDNA was diluted in RNase/DNase free water (1:5). The qPCR reactions were performed using Fast SYBR™ Green Master Mix (ThermoFisher Scientific, Melbourne, Australia) and primers for ribosomal RNA subunits *5.8S* (100% primer efficiency), *18S* (95% primer efficiency), *45S* (100% primer efficiency) and housekeeping Tata box protein (*TBP;*100% primer efficiency) and beta Tubulin (*TUBB;*94% primer efficiency) transcripts. All primer sequences and cycling conditions used in this study are detailed in Supplementary Table S1. Transcript abundance was measured using the CFX384 Touch™ Real Time PCR detection system (Bio-Rad Laboratories Pty., Ltd., Gladesville, Australia). The relative expression of each ribosomal RNA subunit to *TBP* and *TUBB* mRNA was calculated using the ΔΔCT method and presented as a fold change compared to untreated cells.

### Immunofluorescence

Approximately 10,000 fibroblasts were seeded onto 22 × 22 mm coverslips in 6-well plates and incubated for 24 h, prior to transfection. Following transfection, the cells were fixed using acetone:methanol (1:1) on ice for 4 min and air-dried. Fixed cells were washed in PBS containing 1% Triton X-100 to permeabilise the nuclear membrane, and then in PBS to remove excess Triton X-100. Primary antibodies were diluted in PBS containing 0.05% Tween20 and applied to cells for 1 h at room temperature. All antibody details and staining conditions are listed in Supplementary Table S2. Primary antibodies were detected using AlexaFluor488 anti-mouse (cat no. A11001), anti-rabbit (cat. No A11008) or AlexaFluor568 anti-mouse (cat. No A11004) or anti-rabbit (cat. No A11011) (1:400) after incubation for 1 h at room temperature, and counterstained with Hoechst 33342 (Sigma-Aldrich) for nuclei detection (1 mg/ml, diluted 1:125).

### Western Blotting

Cell lysates were prepared with 125 mM Tris/HCl pH 6.8, 15% SDS, 10% Glycerol, 1.25 µM PMSF (Sigma-Aldrich, NSW, Australia) and 1× protease inhibitor cocktail (Sigma-Aldrich) and sonicated 6 times (1 s pulses) before adding bromophenol blue (0.004%) and dithiothreitol (2.5 mM). Samples were heated at 94°C for 5 min, cooled on ice and centrifuged at 14,000 × g for 2 min before loading onto the gel. Total protein (10 µg), measured by BCA, was loaded per sample on a NuPAGE Novex 4–12% BIS/Tris gel (Life Technologies) and separated at 200 V for 55 min. Proteins were transferred onto a Pall Fluorotrans polyvinylidene fluoride (PVDF) membrane at 350 mA for 2 h. Following blocking for 1 h, the membrane was incubated in 5% skim milk powder in 1× TBST containing the primary antibody diluted as shown in Supplementary Table S2. Immunodetection was performed using the Western Breeze Chemiluminescent Immunodetection System (Life Technologies) according to the manufacturer’s instructions. Immunodetection of rabbit conjugated antibodies was performed using a HRP goat anti-rabbit secondary antibody (1:10,000, Dako, Denmark) and the Immobilon Western Chemiluminescent HRP substrate (Merck Millipore, VIC, Australia). Western blot images were captured on a Vilber Lourmat Fusion FX system using Fusion software and Bio- 1D software was used for image analysis.

### Live Cell Imaging

U2OS cells, previously modified to tag the endogenous SFPQ gene with GFP were used for live-cell imaging (Li et al., 2017). Cells were seeded at 1.5 × 10^5^ cells per well in a 12-well plate, 24 h before transfection, with high glucose DMEM (Life Technologies) supplemented with 10% fetal calf serum and 1% penicillin-streptomycin. On the following day, cells in each well were transfected with 100 or 150 nM 2′ *O*-methyl phosphorothioate AOs complexed with Lipofectamine RNAiMAX (Life Technologies) following the manufacturer’s instructions. Immediately after transfection, the plate was transferred to an Incucyte S3 (Essen Bioscience) for live-cell imaging at 1 h intervals under 10 × optical zoom. SFPQ-GFP and activated caspase-3/7 were visualised under standard green and red channels. Incucyte Caspase-3/7 Red Apoptosis Assay Reagent (Essen Bioscience, Cat No. 4704) was used to reveal cell apoptosis events.

### High Resolution Microscopy

SIM imaging was performed using an N-SIM microscope (Nikon Corporation, Tokyo, Japan), with SR Apochromat TIRF 100 × 1.49 NA oil immersion objective. Spherical aberration was reduced using the Ti_2_ automated correction collar at the beginning of the imaging session. Images were acquired using 405 nm, 488 and 561 nm lasers, with stacks of step size 0.12 µm (with top and bottom of samples determined visually), using 3D-SIM mode. Images were reconstructed with NIS Elements software (Nikon Corporation, Tokyo, Japan).

### Transmission Electron Microscopy

Following transfection, cells were washed in PBS and fixed in cold 2.5% phosphate buffered glutaraldehyde overnight. The fixed cells were scraped and centrifuged, then embedded in 4% agarose. The pellets were processed using a Leica tissue processor, moving through 1% aqueous osmium tetroxide, increasing graded alcohols, propylene oxide, propylene oxide/araldite mix, and finally pure araldite resin. The osmicated cells, surrounded by resin in a beem capsule were polymerised overnight at 80°C to form hard blocks. Semi-thin (0.5 micron) sections were taken from the blocks using glass knives, on an RMC ultratome, and stained with methylene blue (0.1% aqueous Methylene Blue with 0.1% Borax) to visualise available cells. Ultrathin sections were then cut from selected blocks, using a Diatome diamond knife, at approximately 95 nm thickness and mounted on copper mesh grids and stained with uranyl acetate (5% uranyl acetate solution in 5% aqueous acetic acid) and Reynold’s lead citrate. The grids were viewed using a JEOL 1400 TEM, at 80 kV and images captured by an 11-megapixel GATAN digital camera at varying magnifications.

### RNA Sequencing and Analysis

RNA quality was confirmed using a Bioanalyser (Perkin Elmar, MA, United States) prior to RNAseq. Samples were sent to the Australian Genome Research Facility (AGRF, Perth and Melbourne, Australia) for whole transcriptome library preparation using the TruSeq Stranded Total RNA Library Prep Kit (Illumina, CA, United States) and ribosomal RNA depletion with the Ribo-Zero-Gold kit (Illumina, CA, United States). Sequencing was performed using an Illumina HiSeq 2500 (Illumina, CA, United States) to generate 100 base pair single end reads, resulting in an average 25 million reads per sample. Raw sequencing files were quality checked using FastQC (0.11.7), with all files passing. No adapter contamination was identified, and reads were not trimmed. Transcript quantification was performed with salmon (0.8.2), using an index constructed from the Ensembl GRCh38.93 annotation. Transcript abundance was summarised to gene-level counts and imported into R using tximport (1.4.0). Differential expression analysis was performed using DESeq2 (1.16.1) and the default parameters (alpha = 0.1). The heatmap of differentially expressed genes (*p*_adj_ < 0.1) was constructed using the heatmap.2 function from R package gplots (3.0.1). Gene ontology analysis was performed using GSEA (3.0) with 1000 gene set permutations. The gene ontology network was constructed using the Enrichment Map plugin for Cytoscape (3.5.1), with cut-offs: *p* < 0.05, FDR <0.1 and edge >0.3. The RNAseq data is available at https://www.ncbi.nlm.nih.gov/geo/query/acc.cgi?acc=GSE121713 (accession number: GSE121713).

### Statistical Analysis

For all PCR and western blot data, averages were used, with error bars displaying the standard error of the mean. An unpaired *t* test was used to determine significance where the Fisher’s exact test showed equal variance between samples. Significance was determined at α = 0.05.

## Results

### Formation of Nuclear Inclusions After Phosphorothioate AO Transfection

There is a significant body of work showing that phosphorothioate AOs can interact non-specifically with intracellular proteins (Crooke et al., 2020) and recruit paraspeckle proteins, forming nuclear inclusions when transfected into Hela cells (Shen W. et al., 2014; Shen et al., 2019). However, the dynamics of the formation of these structures, the long-term consequences on cell physiology, and whether the effects are recapitulated by transfection of 2′ *O*-fully modified phosphorothioate AOs into cells is unknown. In this study, we largely focused on two 2′ *O*-methyl fully modified phosphorothioate AOs, one that promotes *SMN2* exon 7 retention during splicing by targeting the *ISS-N1* intronic silencer motif (Singh et al., 2006) (termed ‘*SMN7D(-10-29)*’) and the other an oligomer sequence reported by Gene Tools LLC and widely used as a transfection control (termed ‘control AO’). Additional AO sequences were also tested in some experiments, as indicated below.

**FIGURE 1:**
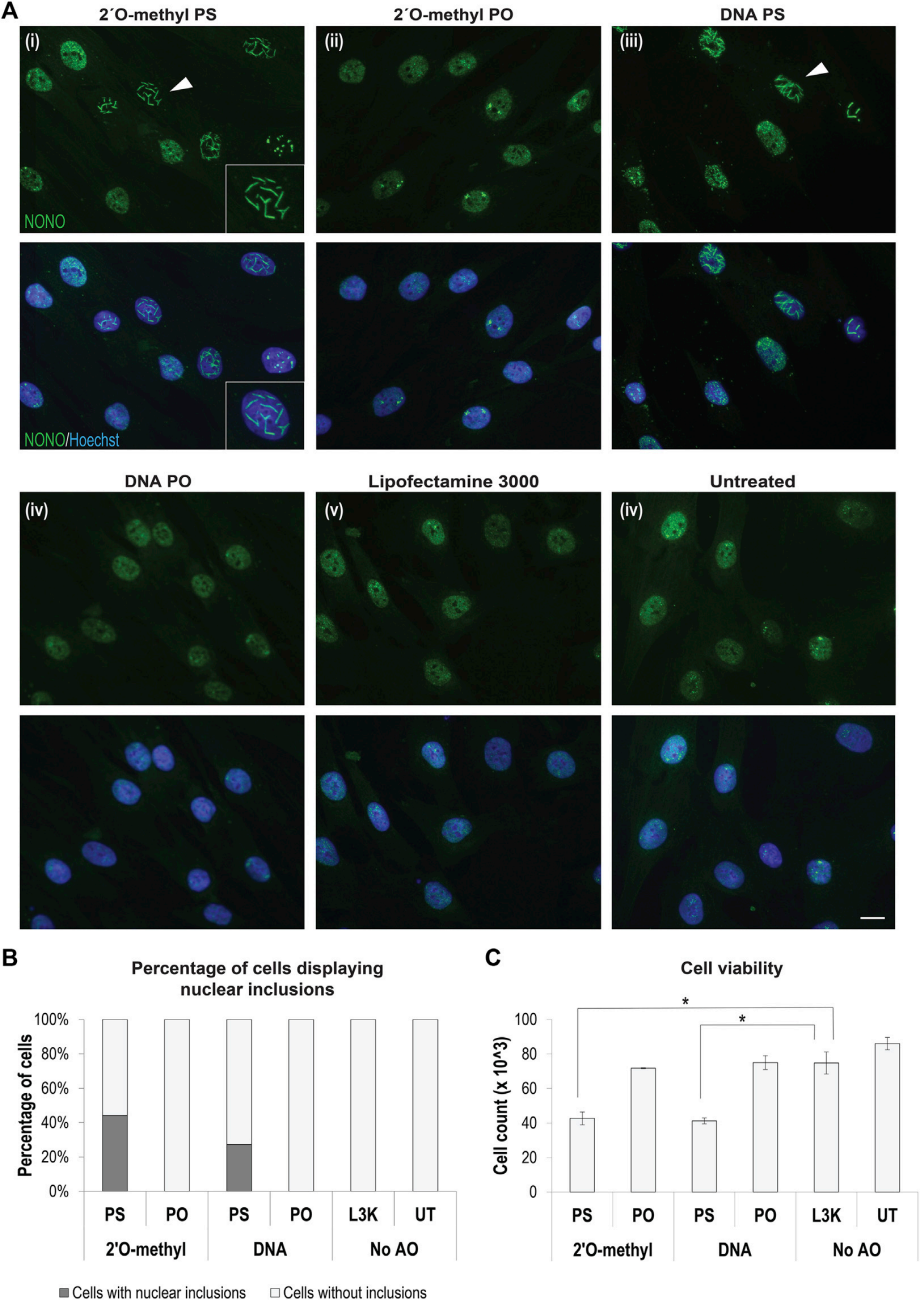
Formation of AO-induced nuclear inclusions associated with cytotoxicity. **(A)** fibroblasts stained for NONO following transfection with different chemically modified antisense oligonucleotides, including representative images of, (i) 2′ *O*-methyl modified bases on a phosphorothioate (PS) backbone, (ii) 2′ *O*-methyl modified bases on a phosphodiester (PO) backbone, (iii) DNA unmodified bases on a PS backbone, (iv) DNA unmodified bases on an unmodified PO backbone, and compared to (v) fibroblasts treated with Lipofectamine 3000 (L3K) or (vi) left untreated. The white arrow indicates nuclear inclusions, magnified within inset (i). Scale bars = 10 μm; **(B)** graph showing the percentage of fibroblasts containing nuclear inclusions following antisense transfection as in **(A)** at 100 nM, with a minimum of 200 cells counted per sample; **(C)** graph showing the number of viable fibroblasts following antisense transfection as in **(A)** (n = 4), error bars represent standard error of the mean and *p*-values were calculated using an unpaired *t*-test, where * denotes *p* < 0.05.

The bulk of studies on gapmer phosphorothioate AO-induced inclusions has been performed in cancer and other immortalized cell lines. We utilized primary human fibroblasts, widely used as patient-specific models, for the majority of our experiments. We transfected fibroblasts with 2′ *O*-methyl fully modified phosphorothioate AOs, fixed the cells 24 h following transfection, and immunostained the cells for the DBHS protein NONO. We observed that 50–70% of cells transfected with fully modified 2′ *O*-methyl phosphorothioate AOs, but not the un-transfected or transfection reagent only (Lipofectamine) treated control cells, developed large nuclear inclusions, identified by aberrant NONO staining (Figures 1A,B). Off-target binding of gapmer AOs by various paraspeckle proteins and the formation of nuclear inclusions, reported by Shen et al. (2015) was found to be phosphorothioate backbone dependent; the extent of the phosphorothioate backbone interaction with DBHS proteins can, in part, be dependent on further AO chemistry modifications. Consistent with the findings by Shen W. et al. (2014), we observed no nuclear inclusions containing NONO in cells transfected with a DNA ribose or 2′ *O*-methyl modified phosphodiester AO but did observe nuclear inclusions in cells transfected with a DNA AO on a phosphorothioate backbone, albeit with half as many cells displaying nuclear inclusions, when compared to the 2′ *O*-methyl modified equivalent, confirming that interaction with DBHS proteins is dependent on the phosphorothioate modification, and exacerbated by the 2′ *O*-methyl modification (Figures 1Ai–iv; Supplementary Figure S1). We next explored any correlation between cell death and the formation of 2′ *O*-methyl phosphorothioate AO-induced nuclear inclusions and observed that transfection with AOs induced nuclear inclusions that correlated with reduced fibroblast survival (Figures 1B,C).

**FIGURE 2:**
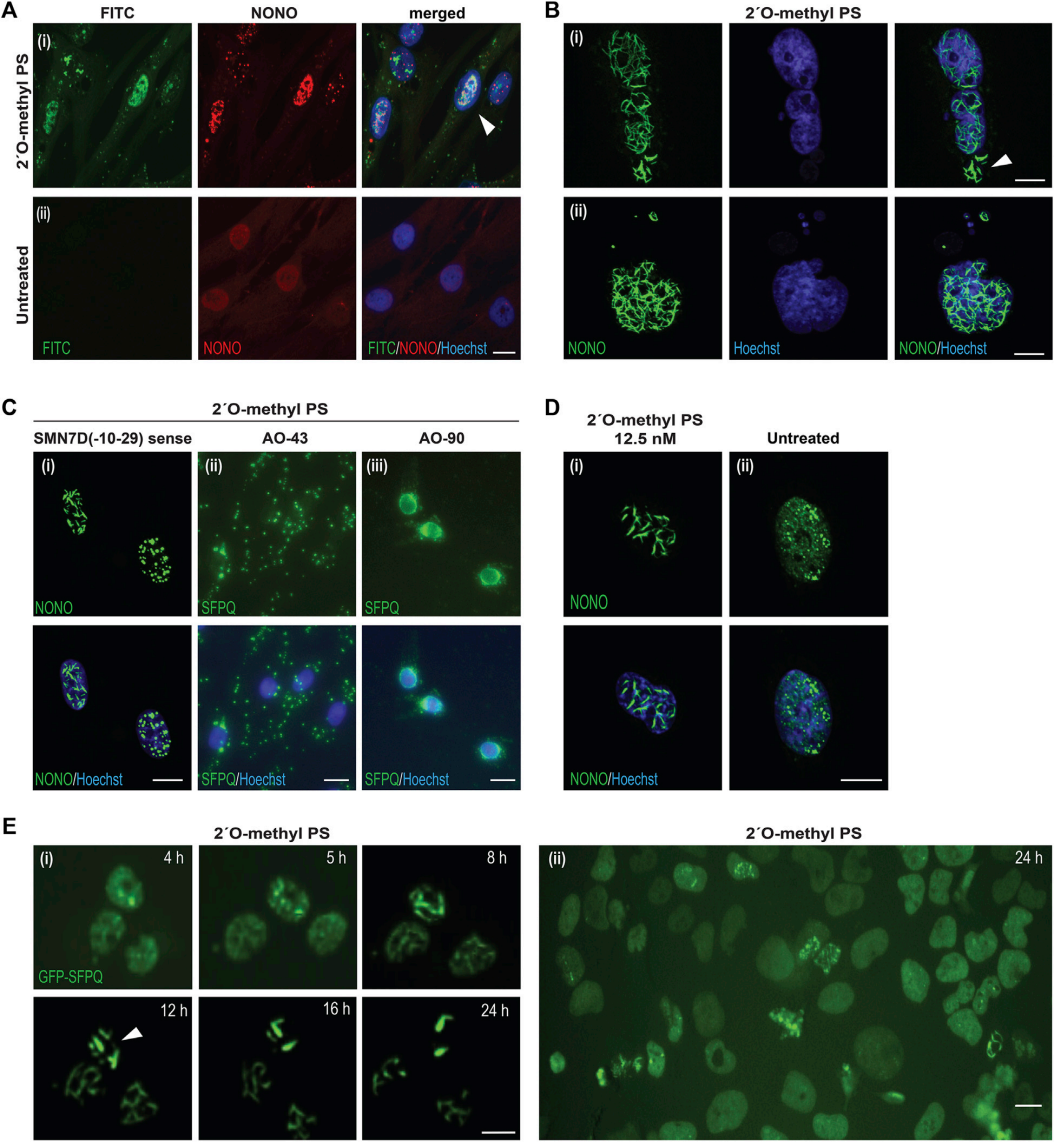
Characterisation of AO-induced nuclear inclusions. **(A)** fibroblasts transfected with a FITC-labelled 2′ *O*-methyl phosphorothioate (PS) AO (100 nM) and co-stained for NONO (i), compared to untreated cells (ii). Colocalization of NONO with the PS-AO is indicated by the white arrow; **(B)** fibroblasts stained for NONO show, (i) nuclear inclusions without evidence of nuclear staining after 2′ *O*-methyl phosphorothioate *SMN7D(-10-29)* transfection, as indicated with the white arrow, (ii) nuclear blebbing after 2′ *O*-methyl phosphorothioate control AO transfection; **(C)** cells transfected with different phosphorothioate AO sequences cause varied patterns of DBHS protein mislocalisation, including: nuclear inclusions of NONO protein in the form of filaments or foci induced by transfection the *SMN7D(-10-29)* sense sequence AO (iii), cytoplasmic aggregation and accumulation of SFPQ protein with AO 43 (Supplementary Table S3), or accumulation of SFPQ around the nuclear envelope following transfection with AO 90 (Supplementary Table S3); **(D)** nuclear inclusions observed in fibroblasts transfected with 12.5 nM of 2′ *O*-methyl phosphorothioate *SMN7D(-10-29)* antisense stained for NONO (i), compared to untreated fibroblasts (ii); **(E)** live cell imaging time-course of U2OS cells expressing endogenous GFP-SFPQ showing the formation of nuclear inclusions over 24 h (i) following 2′ *O*-methyl phosphorothioate control AO transfection (100 nM), inclusions are indicated by the white arrow, with a wider field of view at 24 h displayed in (ii). All scale bars = 10 µm.

To determine the distribution of the phosphorothioate AO within the cell and within the nuclear inclusions, we used a fluorescently labelled FITC-AO to visualize its distribution and interaction with NONO (Figure 2A). Consistent with reports by Shen W. et al. (2014), the phosphorothioate AO did indeed co-localize with NONO within the nucleus, confirming the phosphorothioate AO as the scaffold for NONO positive nuclear inclusions. We also observed that once formed, the inclusions appeared to be highly stable, and remained evident on the culture substrate following cell death and nuclear fragmentation and blebbing (Figures 2Bi,ii).

Others have shown that different modified gapmer phosphorothioate AO sequences induce variable toxicities in cell lines and that this effect is species independent (Shen et al., 2019). To further explore whether AO-induced nuclear inclusions are sequence independent, ninety different 2′ *O*-methyl phosphorothioate AOs (prepared for evaluation in other studies, targeting structural gene transcripts, transcription factors, splicing factors and enzymes), were transfected into fibroblasts at 100 nM and the cells were immunostained for SFPQ (Supplementary Table S3). We found that all AOs tested altered SFPQ distribution, and all but one AO formed nuclear inclusions by 24 h following AO transfection, irrespective of length (18–30 bases) or nucleotide sequence composition (Supplementary Table S3).

The nuclear inclusions appeared as either long ‘filaments’, punctate foci, or both (Figure 2Ci). Others reported cytoplasmic SFPQ aggregates induced by gapmer phosphorothioate AOs, (Liang et al., 2015), in addition to nuclear inclusions; also evident in our cells transfected with a variety of fully modified 2′ *O*-methyl phosphorothioate AO sequences (Figure 2Cii). While most AOs induced nuclear inclusions containing SFPQ, 20 of the AO sequences drew SFPQ from the nucleus to localise within the cytoplasm. Intriguingly, 18 of these AOs, including nucleotide equivalent scrambled sequences with no predicted target binding, had a guanine content of greater than 30%, suggesting sequence specific-effects on protein distribution, with guanine content potentially a driver of DBHS sequestration within the cytoplasm. We further report that one AO targeted SFPQ to the nuclear envelope in transfected cells (Figure 2Ciii). Thus, optimal transfection efficiency of any 2′ *O*-methyl phosphorothioate AO sequence over 18 bases in length is likely to induce either nuclear or cytoplasmic DBHS protein-enriched inclusions.

Although obvious nuclear inclusions were seen in numerous cells transfected at an AO concentration of 100 nM (Figures 1, 2A–C), we also detected evidence of some nuclear inclusions in fibroblasts transfected with AOs at concentrations as low as 12.5 nM, within 24 h following transfection (Figure 2D). Of note, the untreated cells show diffuse nucleoplasmic NONO signal, as well as the expected punctate NONO inside endogenous paraspeckle nuclear bodies (Fox et al., 2018) (e.g., Figure 2Dii). Nuclear inclusions were also observed following 2′ *O*-methyl phosphorothioate AO transfection into normal human myoblasts, transformed mouse *H2K* myoblasts, and the neuroblastoma SH-SY5Y cell line (Supplementary Figure S2).

To assess the dynamics of nuclear inclusion formation, we transfected 2′ *O*-methyl phosphorothioate AOs as lipoplexes (100 nM) into U2OS cells that had been genome engineered to express GFP-labelled SFPQ (Li et al., 2017) and performed live cell imaging (Figure 2E). We observed formation of nuclear inclusions within 5 h, and these intensified over a period of 12 h, by which time all of the microscopically identifiable GFP-SFPQ signal within the nucleus became sequestered into nuclear structures. At the point at which only nuclear inclusions were evident, these coalesced into a smaller number of discrete filaments (Figure 2Ei). Thus, nuclear inclusions form rapidly upon 2′ *O*-methyl phosphorothioate AO transfection. In a larger field view of U2OS cells transfected with the 2′ *O*-methyl phosphorothioate AO we observed that the normally even SFPQ nuclear distribution was clearly disturbed in many cells, forming both filament and foci-like inclusions (Figure 2Eii). Consistent with the reduced cell viability of fibroblasts transfected with the 2′ *O*-methyl AOs, transfection of U2OS cells was also associated with apparently lower cell confluence by 48 h (Supplementary Figure S3), implying either cytotoxicity or growth inhibition in these cells. Transfection of U2OS cells with a DNA phosphodiester AO and appropriate transfection controls did not result in perturbed SFPQ distribution of differences in cell confluence (Supplementary Figure S3), consistent with the findings in fibroblasts. The full time-course videos over the 48-h period are displayed in Supplementary Videos S1–S4. Taken together, we showed that transfection of fully modified 2′ *O*-methyl phosphorothioate AOs is a direct cause of the rapid formation of various DBHS protein inclusions and reduced cell survival. This effect is, to some extent, sequence specific.

### Composition of Nuclear Inclusions Induced by 2′ *O*-methyl Phosphorothioate AO Transfection

The structure and composition of fully modified phosphorothioate AO nuclear inclusions was further investigated by immunostaining of additional selected nuclear proteins that are naturally found in a variety of nuclear structures, such as paraspeckles, the nuclear envelope, nucleoli, nuclear speckles, Cajal bodies and nuclear stress bodies. We observed that the paraspeckle-associated proteins PSPC1 and FUS were present in AO induced nuclear inclusions, as well as FUS accumulating within the cytoplasm (Supplementary Figures S4A,B). In contrast, TDP-43 and hnRNP-A1 were not evident in the nuclear inclusions but also appeared to have greater localization within the cytoplasm when compared to the untreated fibroblasts (Supplementary Figures S4C,D).

**FIGURE 3:**
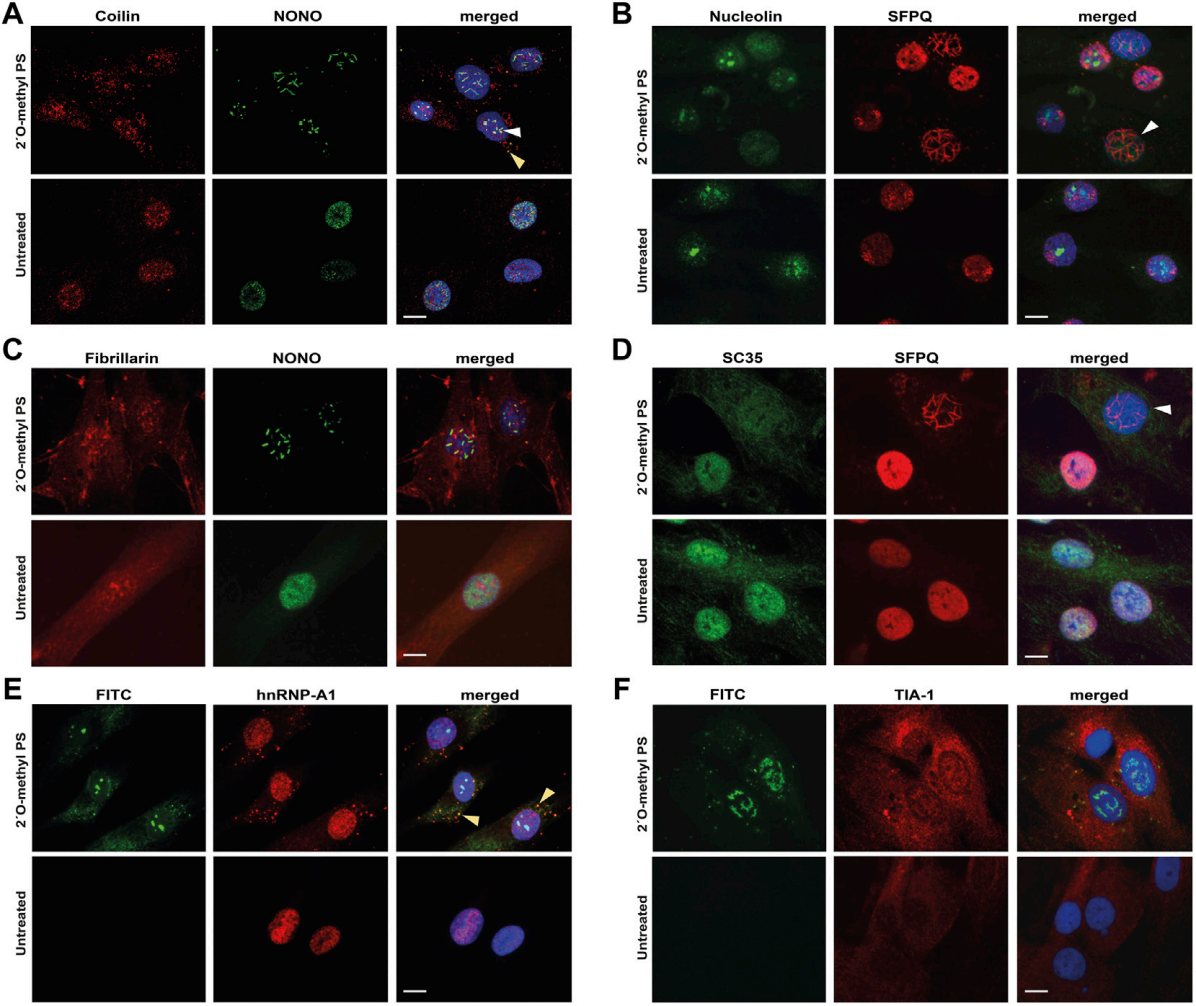
Staining of nuclear and cytoplasmic bodies following 2′ *O*-methyl phosphorothioate (PS) transfection in fibroblasts. Representative images following 2′ *O*-methyl PS AO transfection (100 nM, 24 h) with either the *SMN7D(-10-29)* or the control antisense sequences **(A*–*D)** or the FITC-labelled *SMN7D(-10-34)* sequence **(E*–*F)**, showing: **(A)** staining for Cajal body marker coilin (red) and NONO (green) overlayed with Hoechst, with NONO and coilin colocalization indicated with the arrows within the nucleus (white) and cytoplasm (yellow); **(B)** staining for nucleolar marker nucleolin (green) and SFPQ (red) overlayed with Hoechst (blue), with a cell containing nuclear inclusions and no nucleolar nucleolin staining indicated by the white arrow; **(C)** staining for nucleolar marker fibrillarin (red) and NONO (green) overlayed with Hoechst; **(D)** staining for nuclear speckle marker SC35 (green) and SFPQ (red) overlayed with Hoechst, with a cell containing nuclear inclusions and no nuclear SC35 staining indicated by the white arrow; **(E)** images of FITC fluorescence, overlayed with paraspeckle and stress granule marker hnRNP-A1 (red) and Hoechst, with FITC and hnRNP-A1 colocalization indicated by the yellow arrow; and **(F)** images of FITC fluorescence, overlayed with stress granule marker TIA-1 (red) and Hoechst. All scale bars = 10 µm.

When examining coilin, a component of Cajal bodies, we observed a variety of abnormal staining patterns in 2′ *O*-methyl phosphorothioate AO transfected cells (Figure 3A). Specifically, we saw re-distribution of coilin to the cytoplasm and occasional co-localization within nuclear filaments with NONO, whereas coilin is present almost exclusively in the nucleus, segregated from NONO in untreated cells (Figure 3A). Similarly, both nucleolin and fibrillarin, markers for the nucleolus, showed altered distribution in nuclear inclusion-containing cells (Figures 3B,C) and the splicing factor and component of nuclear speckles, SC35, showed enhanced cytoplasmic localisation and was depleted from the nucleus in cells that also contained nuclear inclusions (Figure 3D). Line intensity profiling shows altered distribution of these nuclear proteins in individual cells that have nuclear inclusions (Supplementary Figure S5).

To further characterize the mechanism underpinning the displacement of nuclear paraspeckle proteins into the cytoplasm, we immunostained fibroblasts for hnRNP-A1, following transfection with the FITC-labeled 2′ *O*-methyl phosphorothioate sequence. Intriguingly, we observed some cytoplasmic co-localization of the AO with hnRNP-A1 in cells that did not appear to form nuclear inclusions (Figure 3E), suggesting that phosphorothioate AOs can indeed interact with nuclear proteins, drawing them to the cytoplasm. The cytoplasmic accumulation of hnRNP-A1 is reminiscent of its distribution within cytoplasmic stress granules, thus, to determine whether phosphorothioate AOs can interact with stress granules, we further immunostained for stress granule marker TIA-1 in FITC-AO transfected cells (Figure 3F). Cytoplasmic TIA-1 is clearly enhanced in 2′ *O*-methyl phosphorothioate transfected cells when compared to untransfected cells, with the appearance of defined stress granules. However, we observed no co-localisation of the FITC labeled AO with TIA-1, suggesting that the AO may not directly interact with the stress granules, and instead, it is possible that the stress of nuclear inclusions and associated toxicity may initiate the stress granule response. The difference in colocalization of the PS-AO between hnRNP-A1 and TIA-1 suggests that the phosphorothioate backbone does not draw hnRNP-A1 into stress granules in the cytoplasm within the 24 h timeframe evaluated.

Since components of subnuclear organelles were disorganized following transfection with phosphorothioate AOs, the levels of these proteins in the transfected cells were investigated. Western blot analysis showed that the overall abundance of each protein studied to be essentially unchanged following phosphorothioate AO transfection. This contrasts with the transfection of 2′fluoro-gapmer phosphorothioate AOs in Hela cells, that resulted in reduced levels of DBHS proteins (Shen et al., 2015). Representative western blots probed for SFPQ, NONO, TDP-43, hnRNP-A1, NCL, HSF1 and beta actin, are shown in Supplementary Figure S6. Thus, 2′ *O*-methyl phosphorothioate AO transfection into fibroblasts results in nuclear inclusions containing paraspeckle proteins, and mis-localisation of markers for other nuclear structures, including nucleoli and splicing speckles.

### Morphology of 2′ *O*-methyl Phosphorothioate AO-Induced Nuclear Inclusions

**FIGURE 4:**
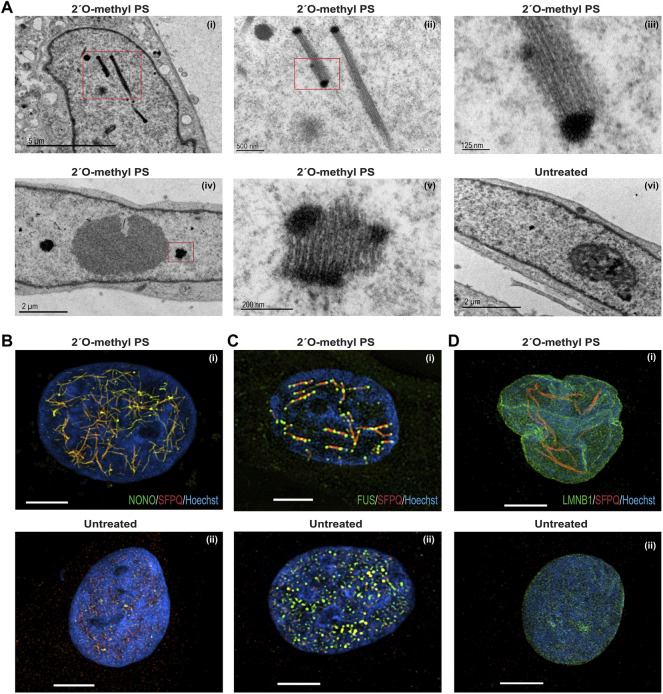
Structural analysis of nuclear inclusions in 2′ *O*-methyl phosphorothioate transfected fibroblasts. Fibroblasts were transfected with the 2′ *O*-methyl phosphorothioate AO, *SMN7D(-10-29)* at 100 nM, for 24 h. Transfected and untreated control cells were processed for transmission electron microscopy **(A)** and super resolution fluorescence microscopy **(B*–*D)**. Showing **(A)** transmission electron microscopy of (i) nucleus with filament-like nuclear inclusions (scale = 5 µm), (ii) higher magnification of (i) (scale = 500 nm), (iii) higher magnification of (ii) showing filament structure (scale = 125 nm), (iv) nucleus with foci-like nuclear inclusions (scale = 2 µm), (v) higher magnification of (iv) (scale = 200 nm), and (vi) untreated nucleus (scale = 2 µm); **(B)** super resolution fluorescence microscopy of SFPQ (red) and NONO (green) co-staining following 2′ *O*-methyl phosphorothioate (*SMN7D(-10-29)*) transfection (i) and an untreated cell (ii); **(C)** super resolution fluorescence microscopy of SFPQ (red) and FUS (green) co-staining following transfection (i) and an untreated cell (ii); **(D)** super resolution fluorescence microscopy of SFPQ (red) and LAMIN-B1 (green) co-staining following transfection (i) and an untreated cell (ii). For all SIM images scale bar = 5 µm.

We next used transmission electron microscopy (TEM) on ultra-thin, osmium-stained sections to carry out detailed ultrastructural analysis of the 2′ *O*-methyl phosphorothioate *SMN7D(-10-29)* AO-induced nuclear inclusions in fibroblasts (Figures 4Ai–vi). We observed that the nuclear inclusions appeared to be microfibrillar or amyloid-like, approximately 200–250 nm in diameter, occurring mostly in groups or bundles. For reference, the width of a DNA double helix is ∼2 nm. If captured in a suitable plane of section, the structures appear to have very electron dense termini. Such nuclear inclusions have not previously been revealed by TEM in the investigating laboratory. The electron dense regions are reminiscent of perichromatin in size and electron density, the inclusions revealed by TEM extend to ∼2,000 nm in length. Numerous small, partially formed inclusions are also evident in nuclei of transfected cells when viewed at higher magnification (data not shown).

Since TEM can only capture segments of the presumed large fibril-like nuclear inclusions, super resolution microscopy was used to examine 2′ *O*-methyl phosphorothioate *SMN7D(-10-29)* AO transfected and untreated fibroblasts (Figures 4B,C). AO transfected fibroblasts co-stained for NONO and SFPQ show a network of many nuclear inclusions characterised by co-localisation of the two proteins, whereas the untreated cells show regular, punctate distribution of the proteins within naturally occurring paraspeckles (Figures 4Bi,ii, respectively). Strikingly, transfected fibroblasts co-stained for SFPQ and FUS reveal large, interconnected fibril-like structures decorated with FUS at mid-points and at the termini, suggesting the electron dense termini seen in the TEM could correspond to these FUS-specific regions (Figure 4Ci). SFPQ, NONO and FUS were all sequestered to the nuclear inclusions, unlike the even nuclear distribution of these proteins in untreated cells (Figure 4Cii).

We next examined the nuclear envelope protein, lamin B1 and observed altered distribution in a proportion of 2′ *O*-methyl phosphorothioate *SMN7D(-10-29)* transfected cells that exhibited nuclear inclusions (Figure 4Di,ii). In these cells, the nuclear envelope labelled by immunostaining of lamin B1 appears multi lobular and distorted, reminiscent of nuclei found in the premature ageing disease Hutchinson-Gilford progeria syndrome and in E145K cells (Taimen et al., 2009). The super resolution image in Figure 4Di clearly illustrates lamin B1 localized in 4 distinct nuclear lobules. In all images (Figures 4B–D), the nuclear inclusions appear to reside between areas that may reflect chromosomal territories. In summary, human fibroblasts with AO-induced nuclear inclusions exhibit striking ultrastructural features, including aberrant nuclear lamina and unusual arrangements of paraspeckle proteins within the inclusions.

### 2′ *O*-methyl Phosphorothioate AOs Affect ribosomal RNA Processing and Maturation

**FIGURE 5:**
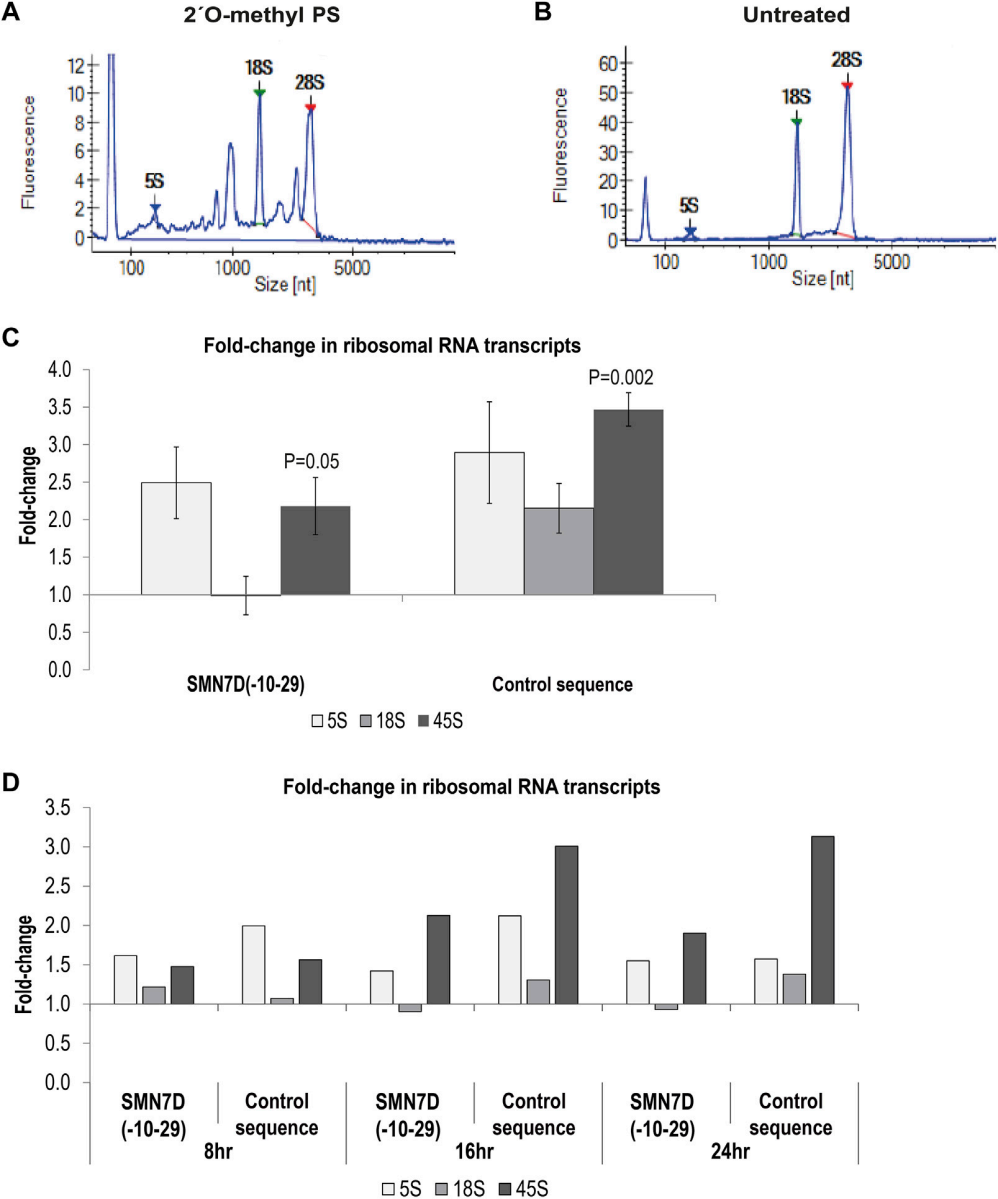
– Analysis of ribosomal RNA processing. Showing bioanalyser trace of rRNA from **(A)** 2′ *O*-methyl phosphorothioate control sequence AO transfected cells, and **(B)** untreated cells; **(C)** qPCR analysis of 5, 18 and 45S rRNA levels following 2′ *O*-methyl phosphorothioate transfection with the *SMN7D(-10-29)* or control AO sequences (24 h). rRNA levels were normalised against *TBP* and compared to those in untreated cells where untreated = 1 (n = 3). Error bars represent the standard error of the mean, and *p*-values were calculated comparing each AO treatment group to the untreated group using an unpaired *t*-test; and **(D)** qPCR analysis of rRNA levels over 8, 16 and 24 h; analysis as in **(C)** (n = 1).

Others have shown that some gapmer phosphorothioate AO sequences cause toxicity through sequestration of DBHS proteins to nucleoli and disrupted *5.8S* RNA metabolism (Shen et al., 2019) [for review see (Crooke et al., 2020)]. Since Cajal bodies and fibrillarin, disrupted in our study, are also important in ribosomal RNA processing and assembly, we assessed rRNA levels in 2′ *O*-methyl phosphorothioate AO transfected cells and untreated cells. qPCR and bioanalyser data showed that unprocessed rRNA was markedly increased by 2′ *O*-methyl phosphorothioate AO transfection in fibroblasts (Figure 5). A Bioanalyser RNA trace from 2′ *O*-methyl phosphorothioate AO transfected fibroblasts (Figure 5A) showed low levels of *18S* and *28S* ribosomal subunits, as well as a several intermediate peaks, not present in RNA from untreated fibroblasts (Figure 5B), consistent with incomplete rRNA processing in the transfected cells. We ruled out that this effect was due to poor RNA quality, as a similar pattern was observed in all experimental repeats (n = 6) and that RNA integrity numbers (RIN) were measured at 10 for all samples from untreated, DNA transfected and lipofectamine treated cells, yet RINs of 6.4–8.8 were measured for the RNA collected from phosphorothioate AO treated cells, with all samples processed equally. This observation was supported by qPCR analysis of rRNA levels showing a 2–3.5-fold increase in the level of the unprocessed *45S* rRNA in 2′ *O*-methyl phosphorothioate transfected compared to untreated fibroblasts (Figure 5C). Unprocessed rRNA accumulated over time, following the formation of nuclear inclusions (Figure 5D). Thus, fully modified phosphorothioate AOs that form nuclear inclusions sequestering DBHS proteins can also disrupt rRNA metabolism, without the involvement of RNase H1.

### 2′ *O*-methyl Phosphorothioate AO Transfection Promotes Apoptosis

**FIGURE 6:**
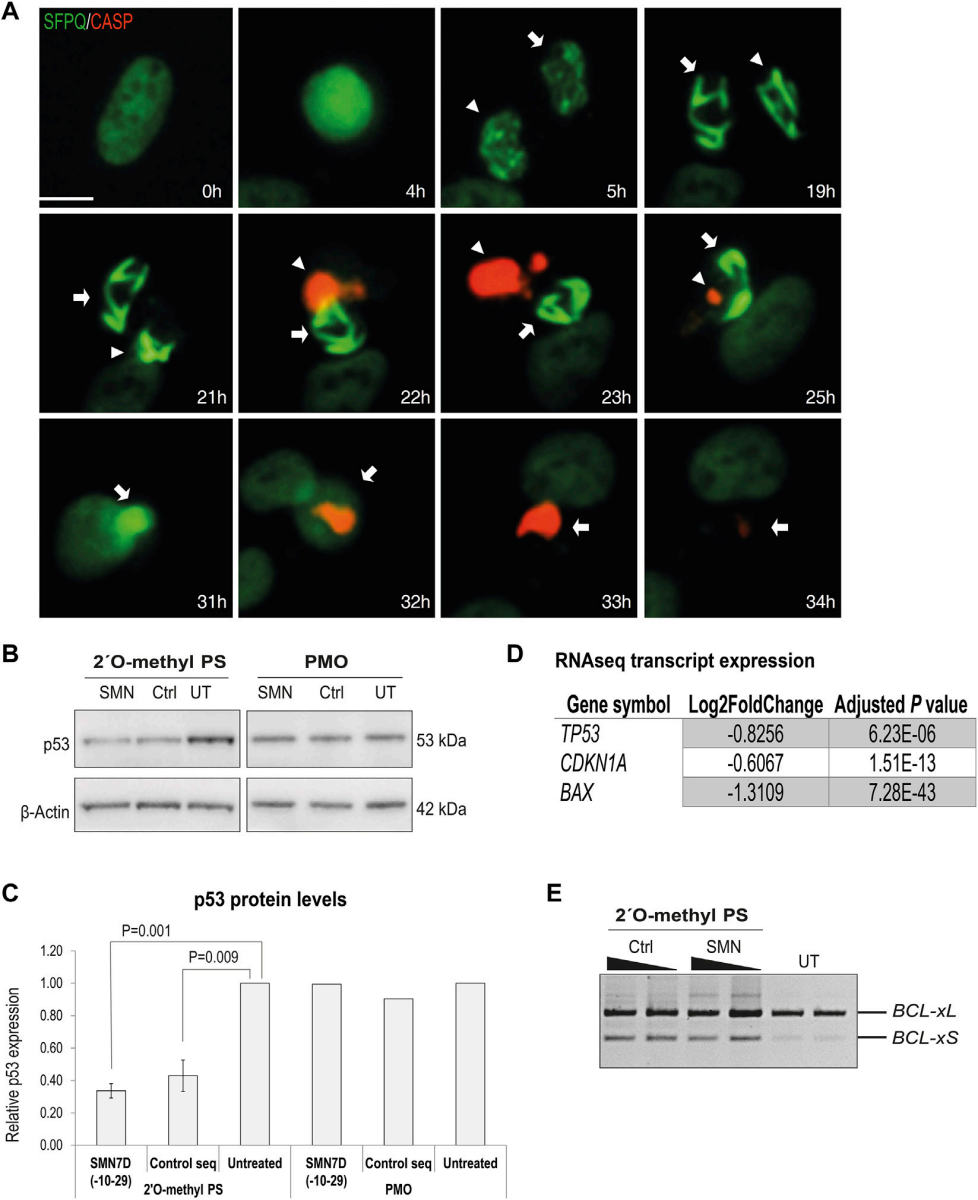
Analysis of cellular toxicity following 2′ *O*-methyl phosphorothioate AO transfection. **(A)** live cell imaging over time of U2OS cells with endogenous GFP-SFPQ (green) transfected with the 2′ *O*-methyl phosphorothioate control sequence and stained for caspase 3/7 activation (red). Two sister cells are annotated with arrows, and scale bar = 10 μm; **(B)** western blot of p53 and β-actin levels in fibroblasts following 2′ *O*-methyl phosphorothioate AO (n = 3) and PMO transfection (n = 1); **(C)** densitometry analysis of **(B)** where p53 levels are normalized to β-actin and compared to those in untreated cells where untreated = 1. Error bars represent the standard error of the mean and *p*-values were calculated using an unpaired *t*-test; **(D)** table showing log2Fold-change in expression of key transcripts involved in p53 signaling pathway from RNAseq analysis following 2′ *O*-methyl phosphorothioate AO transfection; and **(E)** RT-PCR analysis of *BCL2* transcripts in fibroblasts following 2′*O*-methyl phosphorothioate AO transfection, as indicated at 100 and 50 nM (24 h).

Following phosphorothioate AO transfection of GFP-SFPQ expressing U2OS cells (as in Figure 2) at 100 nM, live cell imaging to evaluate caspase activation was undertaken using a caspase-sensitive dye (Figure 6A). Focusing on individual cells with inclusions, we observed nuclear inclusions in two sister cells, 5 h after transfection. Nuclear inclusions became larger and more compact over time, followed by caspase activation, 22 h after transfection. No caspase activation was observed in the absence of intranuclear inclusions, suggesting that the presence of the inclusions was complicit in caspase activated apoptosis, leading to cell death. Others have shown when using a 2′ fluoro-modified phosphorothioate AO in mouse cells that cell death was tumour suppressor protein p53 dependent, although some p53-independent mechanisms also played a role (Shen et al., 2018). We therefore carried out western blotting of protein extracts from fibroblasts transfected with 2′ *O*-methyl phosphorothioate AOs, using antibodies against p53. We observed differently in this setting, in that p53 was decreased in transfected cells, relative to that in untreated control cells (Figures 6B,C). RT-PCR of *BCL2*-like isoforms also indicated an increase in the shorter, pro-apoptotic isoform in the 2′ *O*-methyl phosphorothioate AO transfected fibroblasts, compared to untransfected cells, again suggesting apoptosis as a result of the AO transfection (Figure 6E). As a comparison, we also measured p53 levels following transfection of PMOs of the same sequences and noted that p53 levels remained unchanged relative to those in untreated cells (Figures 6B,C). To further support the finding that fully modified phosphorothioate AOs suppressed p53 in primary cells, we investigated the expression of a number of p53 regulated transcripts following RNAseq analysis (Figure 6D). The p53 encoding transcript *TP53* was also significantly downregulated in the RNAseq dataset, along with the p21 encoding transcript *CDKN1A* and *BAX*, two critical players in the p53 signaling pathway, supporting that in the primary cell model evaluated, caspase activation following fully modified phosphorothioate AO transfection was p53 independent.

### Transcriptome Analysis of 2′ *O*-methyl Phosphorothioate Transfected Cells Reveals Global Cellular Disruptions

**FIGURE 7:**
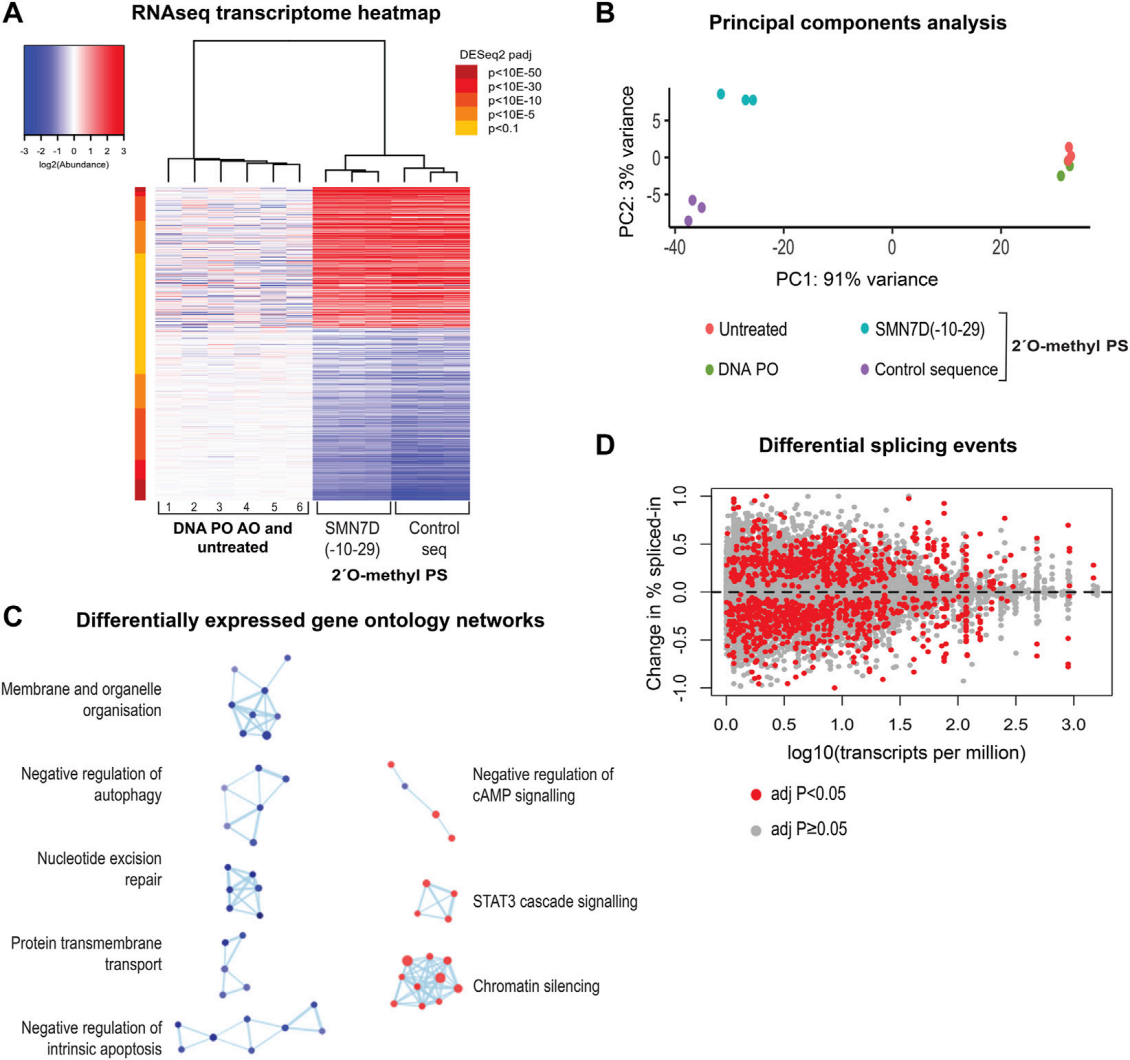
RNA sequencing analysis of 2′ *O*-methyl phosphorothioate AO transfected fibroblasts. **(A)** heatmap of all differentially expressed transcripts in control (1, 4, 5-untreated and 2, 3, 6, DNA PO AO) and 2′ *O*-methyl phosphorothioate AO *SMN7D(-10-29)* and control AO sequence transfected (100 nM, 24 h) cells, whereby red represents overexpressed transcripts, and blue represents under-expressed transcripts; **(B)** principal components analysis of RNA profile between groups; **(C)** gene ontology (GO) network of significantly affected cellular pathways in samples from transfected, compared to untreated and DNA AO transfected cells. GO terms are represented as dots being either overexpressed (red) or under-expressed (blue), and groups with greater than 50% of genes in common are linked; and **(D)** differential changes in splicing displaying the change in percent spliced in events against total transcript expression as transcripts per million.

We next evaluated the effect of 2′ *O*-methyl phosphorothioate AO transfection and nuclear inclusion formation on the transcriptome by RNAseq. We prepared RNA from fibroblasts transfected with the two 2′ *O*-methyl phosphorothioate AOs used throughout this study, as well as from untreated cells and cells transfected with a DNA phosphodiester AO lipoplex (100 nM for 24 h) for RNAseq analysis (n = 3). A heatmap comparing the expression of transcripts in 2′ *O*-methyl phosphorothioate AO transfected fibroblasts to that in cells transfected with the DNA phosphodiester oligonucleotide and untreated cells showed stark differences in expression between the groups (Figure 7A). A principal components analysis (PCA) clustered untreated and the DNA AO transfected cells perfectly, indicating that neither the lipofectamine transfection reagent or the DNA AO elicited alterations to the transcriptome (Figure 7B). A striking 91% variance was observed in the PCA between the two phosphorothioate AO transfected sample groups and the untreated and DNA-AO transfected groups, while only 3% variance in transcript profiles was observed between the two phosphorothioate AO transfected groups (Figure 7B).

Gene ontology (GO) analyses showed significant disruptions to major cellular pathways. The GO network (Figure 7C) revealed components of signaling pathways, chromatin silencing, and metabolic pathways to be over-represented, while pathways regulating both apoptosis and autophagy, membrane and organelle organisation, protein transmembrane transport and nucleotide excision repair components are under-expressed. Gene ontology pathways included in each cellular process are listed in Supplementary Table S4. Overall, these data show dramatic and widespread changes to gene expression in human fibroblasts, resulting from uptake of the 2′ *O*-methyl phosphorothioate AOs.

Given that the use of fully-modified AOs is common practice in developing splice-switching AOs, and that many paraspeckle proteins play a critical role in the splicing process, it was necessary to investigate changes in splicing events as a result of phosphorothioate AO transfection and protein interaction. Figure 7D illustrates differential splicing events as determined by RNAseq analysis, whereby the fold-change in the percentage spliced-in is plotted against transcript expression. We observed a notable difference in both increased and decreased exon retention events compared to baseline, as indicated by either positive or negative plots, respectively, suggesting global changes in pre-mRNA splicing.

Global changes to the transcriptome following phosphorothioate AO transfection are evident, however the extent and consequences of deleterious effects are unknown. Table 1 shows key genes involved in critical cellular processes that were differentially expressed by a minimum Log_2_FoldChange of ±0.7 (*p*_adj_ < 0.05) in fibroblasts following AO transfection. Transcripts encoding key paraspeckle protein NONO (*Log2FoldChange −1.6, p = 1.33E-134*) as well as for hnRNP-A1 (*−0.89, p = 1.02E-89*) were observed to be downregulated, while *SFPQ* was only modestly downregulated (*−0.25, p = 2.5E-10*) and *PSPC1* modestly upregulated (*0.55, p = 1.73E-5*). Interesting, the long non-coding RNA *NEAT1*, essential for paraspeckle formation and displaced by the phosphorothioate AO interaction, was modestly increased (*0.11, p = 0.026*), while *MALAT1* (*NEAT2*) was significantly upregulated (*1.36, p = 1.6E-50*). Given the observed changes in splicing events (Figure 7D), we highlight a number of transcripts critical in determining pre-mRNA splicing events, such as those involved in forming the spliceosome as well as major splicing factor families, including those encoding the serine/arginine splicing factor (SRSF) proteins, heterogeneous ribonuclear proteins (hnRNP) and the pre-mRNA processing factor (PRPF) proteins.

**TABLE 1:** Select genes that are significantly differentially expressed following transfection with phosphorothioate AOs. All with Log2FoldChange of ±0.7and *p*_adj_ < 0.05.

Gene function	Key gene transcripts downregulated	Key gene transcripts upregulated
Paraspeckle formation	*NONO, HNRNPA1*	
Long non-coding RNAs		*MALAT1*
Housekeeping	*ACTB, GAPDH, LMNA, TUBB*	*CCND1*
ALS/neurodegenerative disease linked genes	*TARDBP, SOD1, SQSTM1, NEK1, HNRNPA1, VCP*	
Regulation of autophagy	*BECN1, MAP1LC3B2, LAMP2, ATG4A/B/C*	
Mitochondrial genes	*PINK1, OMA1, TOMM7, TOMM20, OPA1, PRDX3, NDUFS1*	
Chromatin regulators and remodelers	*CHD3, CHD4 CHD6, BRD7, RSF1, PRMT2, ATM*	*CHD2, CHD5, CHD7*
SRSF splicing factor family	*SRSF5, SRSF9, SRSF11*	*SRSF7, SRSF12*
hnRNP splicing factor family	*HNRNPA0/1/3, HNRNPC, HNRNPD, HNRNPH1/2, HNRNPK, HNRNPM, HNRNPU*	
PRPF splicing family	*PRPF6, PRPF8, PRPF19, PRPF31, PRPF38A, PRPF40 A/B*	
HDAC family	*HDAC1, HDAC2, HDAC5, HDAD6, HDAC7, HDAC8, HDAC10, HDAD11*	*HDAC9*
Transcription factors	*TP53, SP1, SUPT3H, SUPT4H1, SUPT5H, SUPT7L*	*MYC, E2F1*
Stress markers	*NPM1, FBL, TIA1, G3BP1*	*NOP2*

Of critical relevance to the use of AOs in treating neurodegenerative diseases and proteinopathies, we draw attention to transcripts encoding proteins involved in autophagy regulation as well as those linked to neurodegenerative diseases, such as ALS. Other protein families that were significantly dysregulated at the transcript level included those involved in transcription e.g. the SUPT proteins, mitochondrial function, chromatin structure and epigenetic regulation, e.g. the histone deacetylases (HDAC). Consistent with nucleolar disruption and mis-regulation of ribosomal RNA, key nucleolar stress markers were also dysregulated, including those encoding p53, c-Myc, E2F-1 and SP1 transcription factors (Yang et al., 2018). Heatmaps showing altered expression of transcripts regulated by these factors are shown in Supplementary Figure S7. Of particular concern for all studies evaluating phosphorothioate AOs, we observed that commonly utilized housekeeping genes, including those encoding β-actin, glyceraldehyde 3-phosphate dehydrogenase (GAPDH), lamin-A and β-tubulin were all downregulated, while cyclin-D was upregulated, suggesting caution be taken when normalizing expression to these transcripts. An expanded list of these transcripts, and the Log2FoldChange and *p*-value, is included in Supplementary Table S5.

### PMOs, or Double-Stranded 2′ *O*-methyl Phosphorothioate AOs, Do Not Form Nuclear Inclusions, Protecting From Cellular Toxicity and Off-Target Transcript Effects

**FIGURE 8:**
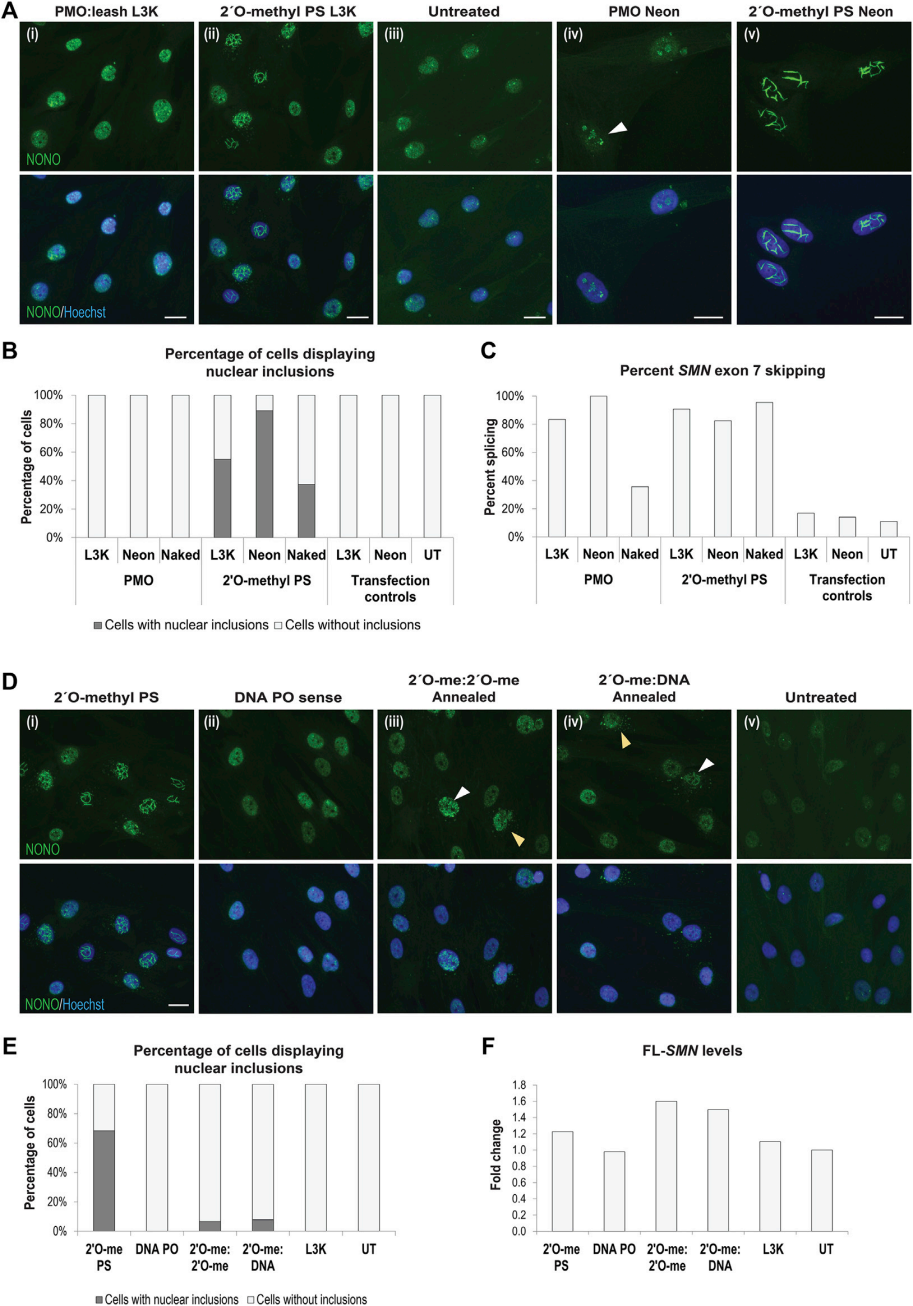
AO chemistries that do not cause nuclear inclusions. **(A)** representative images of normal fibroblasts stained for NONO after transfection with (i) PMO *SMN7A(+13+32)* using a complementary DNA leash and lipofectamine 3000 (L3K) transfection reagent (100 nM, 24 h), compared to (ii) 2′ *O*-methyl phosphorothioate (PS) *SMN7A(+13+32)* transfected using L3K; (iii) untreated cells; and higher magnification representative images of (iv) PMO *SMN7A(+13+32)* transfected using Neon electroporation (50 μM, 24 h), with nucleolar NONO staining indicated with the white arrow, compared to (v) 2′ *O*-methyl PS *SMN7A(+13+32)* transfected using Neon electroporation (50 µM); **(B)** graph displaying the number of cells containing nuclear inclusions, as in **(A)**, following PMO and 2′ *O*-methyl PS AO transfection using L3K (100 nM), Neon electroporation (50 µM) and uncomplexed ‘naked’ transfection (10 µM), with a minimum of 200 cells counted per treatment; **(C)** graph displaying the changes in *SMN* exon 7 splicing following transfection as in **(B)**; **(D)** representative images of SMA fibroblasts stained for NONO following transfection with the *SMN7D(-10-29)* (i) 2′ *O*-methyl phosphorothioate AO (2′O-me PS), (ii) the complementary DNA leash AO sequence (DNA PO), (iii) double stranded annealed antisense and sense 2′ *O*-methyl PS AOs (2′ O-me:2′ O-me, 100 nM of each AO), and (iv) double stranded annealed 2′ *O*-methyl PS antisense and DNA PO sense leash (2′ O-me:DNA, 100 nM of each AO), compared to (v) untreated cells; **(E)** graph displaying the number of cells containing nuclear inclusions for the experiments in **(D)**, with a minimum of 200 cells counted per treatment; **(F)** graph displaying changes in full length SMN (FL-*SMN*) expression, normalised to TBP and presented as a fold-change compared to untreated SMA cells, following transfection as in **(D,E)**. All scale bars = 10 µm.

While transfection of gapmer phosphorothioate AOs resulted in nuclear inclusions (Shen W. et al., 2014), PMOs are yet to be evaluated in this context. We therefore transfected fibroblasts with the AO sequences described above, synthesized as PMOs, and carried out immunofluorescence against NONO. Transfection of the PMO sequences into fibroblasts did not alter the apparent distribution of NONO, nor of any other nuclear proteins studied, when delivered using the Lipofectamine transfection reagent (Figures 8A,B). In order to deliver the PMO using the equivalent concentration and delivery technique to the 2′ *O*-methyl phosphorothioate AO, the PMO was annealed to a complementary DNA ‘leash’ and transfected as a lipoplex (100 nM, 24 h). While no altered NONO distribution was observed with the PMO, clear NONO positive nuclear inclusions were observed in the 2′ *O*-methyl transfected fibroblasts in the same experiment (Figure 8Aii). To further demonstrate robust cellular uptake of these PMOs, we used two additional established delivery techniques: Neon electroporation (50 and 10 µM), incubated for 24 h, and un-complexed ‘naked’ PMO transfection, relying on gymnotic uptake (10 µM), incubated for 72 h (Figures 8A,B). Immunofluorescent detection of NONO showed no PMO-induced nuclear inclusion formation following transfection, under any of the conditions used (Figures 8A,B), and was reproducible, irrespective of the PMO sequence (not shown). Interestingly, a proportion of the fibroblasts transfected with 50 µM of the PMO by Neon electroporation showed NONO distribution within the nucleolus at 24 h after transfection (Figure 8Aiv), yet this was not associated with nuclear inclusions, as observed in fibroblasts transfected with the 2′ *O*-methyl phosphorothioate AOs at the same concentration, with 90% of cells containing nuclear inclusions (Figure 8Av). Efficiency of transfection with *SMN7A(+13+32)* AO sequences and induced exon 7 skipping in the target *SMN* transcripts was assessed by RT-PCR across *SMN* exons 4-8, confirming that the absence of intranuclear inclusions in PMO transfected cells occurred despite robust cellular uptake of the PMO using each of the transfection techniques (Figure 8C; Supplementary Figure S8). The PMO chemistry is therefore a promising alternative to 2′ *O*-methyl phosphorothioate AOs for avoidance of nuclear inclusions in splice-switching applications.

We hypothesized that nuclear inclusion formation may be circumvented by delivery of double stranded AOs formed by annealing two complimentary fully modified phosphorothioate AOs. We therefore evaluated several 2′ *O*-methyl phosphorothioate AOs, transfected either individually (Figure 8Di) or annealed using a decreasing thermal incubation protocol (Figure 8Diii). Different AO sequences were transfected in this manner and the cells were immuno-stained for SFPQ or NONO to identify any nuclear inclusion formation, with representative NONO staining images from the *SMN7D(-10-29)* sequence transfected cells shown in Figure 8D, with the percentage of cells containing nuclear inclusions shown in Figure 8E. Complementary 2′ *O*-methyl phosphorothioate antisense and sense AO sequences, delivered individually, both formed nuclear inclusions in up to 68% of cells (Figure 8Di). Of note, while the antisense sequence formed NONO-filaments (Figure 8Di), the sense sequence predominantly sequestered NONO into foci, as shown in Figure 2Ci, supporting our other observations of sequence-specific effects on the phosphorothioate AO-protein interactions. Annealing equal amounts of the two complementary AOs prior to transfection appears to abrogate NONO sequestration, with only 7% of cells showing inclusions after transfection with 100 nM of annealed AO duplex (Figure 8Diii), and no cells showing inclusions after transfection at 50 nM of the AO duplex (not shown). Cytoplasmic distribution of NONO was observed in a similar percentage of cells, however, diffuse NONO staining was also observed within the nucleus of these cells, suggesting that NONO was not entirely drawn from the nucleus, as with certain 2′ *O*-methyl phosphorothioate AOs. We further determined that the annealing of a DNA sense AO, much like a leash, to a fully modified antisense phosphorothioate 2′ *O*-methyl AO has a similar protective effect in preventing protein interaction and nuclear inclusions. Using the *SMN7D(-10-29)* sequence transfected into SMA patient fibroblasts, 8% of cells transfected with the 2′ *O*-methyl phosphorothioate:DNA hybrid contained nuclear inclusions (Figure 8Div). NONO was evident in the cytoplasm in some cells at a similar rate to that induced by the annealed 2′ *O*-methyl phosphorothioate AOs. Densitometry analysis of RT-PCR of *SMN* transcripts confirmed robust transfection efficiency, with the annealed all-modified phosphorothioate duplex and the 2′ *O*-methyl phosphorothioate:DNA hybrid duplex proving equally effective at inducing *SMN2* exon 7 retention as the 2′ *O*-methyl phosphorothioate AO alone (Figure 8F; Supplementary Figure S8). The *SMN7D(−10–29)* AO is designed to reinforce exon 7 retention of *SMN2* in SMA, increasing the production of full-length SMN (FL-*SMN*). Intriguingly, when examining total FL-*SMN* expression (Figure 8F), the annealed AOs induced up to 1.6- and 1.5-fold increase in FL-SMN with the 2′ *O*-methyl:2′ *O*-methyl and the 2′ *O*-methyl:DNA, respectively, compared to that of untransfected SMA fibroblasts, while the 2′ *O*-methyl phosphorothioate sequence alone only induced a 1.2-fold increase in FL-*SMN*, suggesting that antisense activity is in fact improved by delivering phosphorothioate AOs as double stranded molecules. Thus, pre-annealing complementary 2′ *O*-methyl phosphorothioate AOs appears to abolish the tendency to induce DBHS-protein enriched inclusions, presumably increasing the bioavailability and activity of the AO.

**FIGURE 9:**
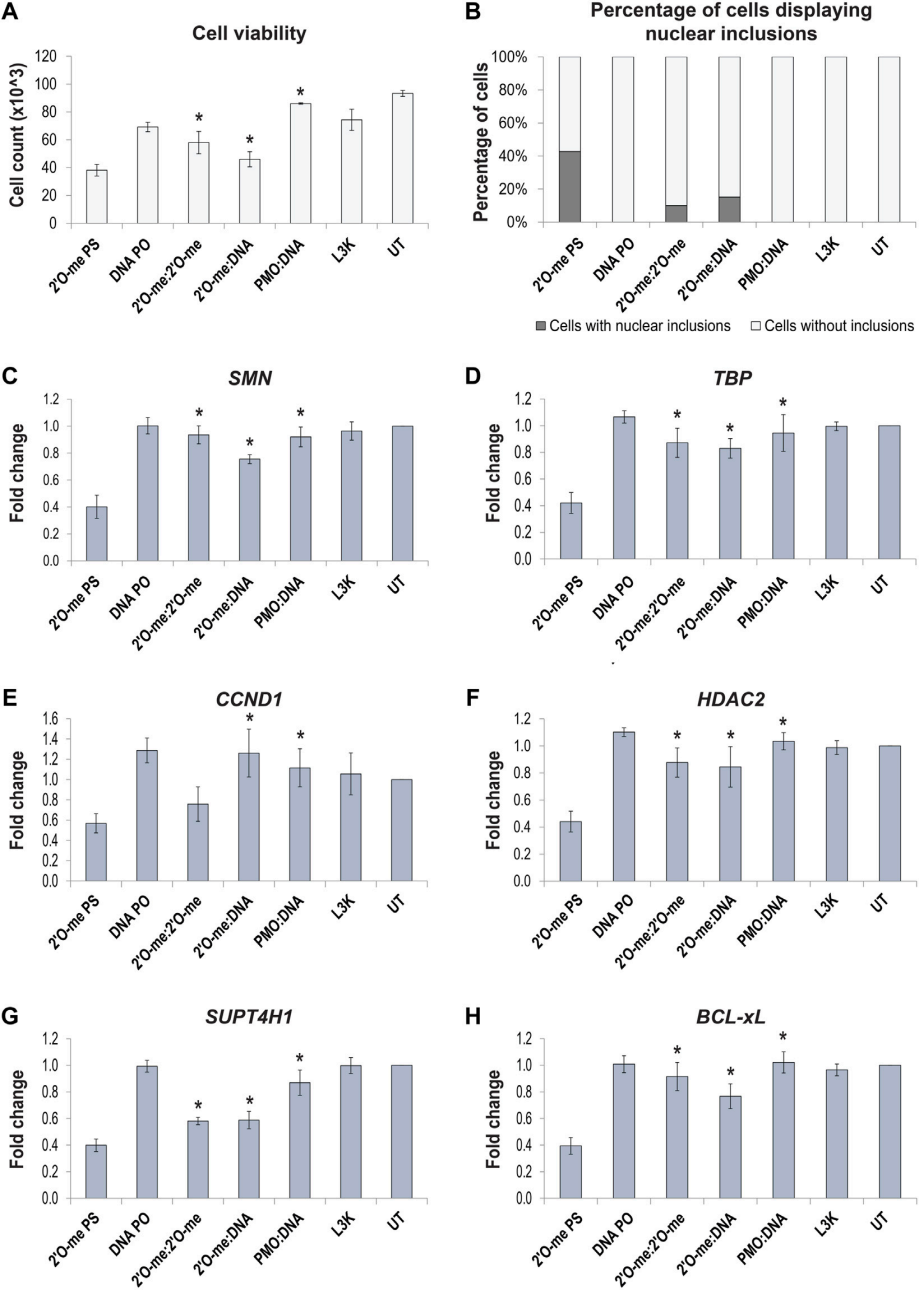
Effect of 2′ *O*-methyl phosphorothioate (2′O-me PS) AO induced nuclear inclusions on cell viability and off-target transcript expression following transfection of normal fibroblasts with AO sequences as different chemistries. **(A)** graph showing the average number of viable fibroblasts following lipofectamine 3000 (L3K) transfection with the *SMN7D(-10-29)* antisense and sense sequences, including 2′ O-me PS and DNA phosphodiester (PO) single stranded AOs and double stranded antisense/sense 2′ O-me:2′ O-me, 2′ O-me:DNA and PMO:DNA AOs, compared to the L3K and untreated (UT) transfection controls (n = 4); **(B)** graph displaying the number of cells containing nuclear inclusions, with a minimum of 200 cells counted per treatment; **(C*–*H)** graphs showing the RT-PCR analysis (densitometry) of selected transcript expression following AO transfection, including **(C)** total *SMN* transcripts, **(D)** *TBP*, **(E)** *CCND1*, **(F)** *HDAC2*, **(G)** *SUPT4H1* and **(H)** *BCL-xL*, (n = 4), error bars represent the standard error of the mean. *p*-values were calculated comparing to the 2′ *O*-methyl phosphorothioate transfected samples using an unpaired *t*-test, where * denotes *p* < 0.05.

To determine whether preventing formation of DBHS protein positive nuclear inclusions by way of annealing complementary AOs or utilizing PMOs can prevent the downstream off-target effects and cellular toxicity, we measured cell viability and transcript expression of selected transcripts, shown to be dysregulated in the RNAseq analysis. Fibroblasts were transfected with three different 2′ *O*-methyl phosphorothioate AO sequences and corresponding 2′ *O*-methyl:2′ *O*-methyl fully phosphorothioate modified complex, 2′ *O*-methyl phosphorothioate:DNA hybrid and a PMO:DNA (leash) hybrid. Interesting, cell viability (Figure 9A) did not entirely correlate with the percentage of cells displaying nuclear inclusions (Figure 9B) in transfected cells. However, we did observe a significant increase in the viability of cells transfected with the annealed AOs, with both complementary, fully modified and DNA AOs, and with PMOs transfected using a leash. The PMO transfected cells showed the highest survival after transfection under all the conditions evaluated. AO-mediated effects on transcript expression were analyzed by assessing *SMN* splicing by densitometry analysis of RT-PCR across exons 4–8; *TBP* levels as a housekeeping/loading control, *CCND1*, *HDAC2* and *SUPT4H1* transcripts shown to be dysregulated by RNAseq, and *BCL-xL* shown to be decreased in our study (Figures 9C–H, respectively). Gel images of RT-PCRs are shown in Supplementary Figure S9. Interestingly, we observed each of these transcripts to be significantly downregulated following 2′ *O*-methyl phosphorothioate transfection, including *TBP* that was not dysregulated according to the RNAseq data. As such, we were unable to use *TBP* as a loading control for normalization and given that equal amounts of RNA were used for the RT-PCRs, with three different 2′ *O*-methyl phosphorothioate sequences evaluated (n = 4), we instead determined the fold-change in raw transcript expression between transfected and untreated samples. Expression of each of the transcripts evaluated was corrected to varying degrees by use of the double stranded annealed AOs and PMOs. The levels of transcript correction correlated with the percentage of cells containing nuclear inclusions, as well as improved cell viability, suggesting that the use of double stranded AOs or PMOs can overcome the propensity to form nuclear inclusions, as well as the toxic and off-target effects of phosphorothioate AOs.

## Discussion

Antisense oligonucleotides have therapeutic potential as modulators of gene expression in many different diseases and can do so through several different mechanisms. Antisense drugs that are in current clinical use either alter exon selection during pre-mRNA processing, or induce RNase H1 degradation of the target mRNA, the mechanism of action determined by the nature of the AO chemistry (Le et al., 2016). However, emerging reports of off-target effects conferred by gapmer phosphorothioate AOs, identified primarily in cancer cell lines *in vitro* (Shen W. et al., 2014; Shen et al., 2015) and *in vivo* (Shen L. et al., 2014; Shen et al., 2018) prompt careful examination of the molecular effects of these compounds. Moreover, the off-target effects may also represent an opportunity for gaining structural insights into protein-AO inclusions. Here, we primarily use cultured human dermal fibroblasts to characterize how transfection of 2′ *O*-methyl fully modified phosphorothioate AOs cause large nuclear inclusions, with key structural features. We show evidence that 2′ *O*-methyl phosphorothioate AO transfection alters the distribution of proteins normally associated with subnuclear bodies, impacts on global transcript expression and ribosomal RNA processing, among other critical cellular processes, and induces cell stress, followed by apoptosis.

The off-target effects (Shen W. et al., 2014; Shen et al., 2015; Shen et al., 2017) and non-specific binding of proteins, initiated by the negatively charged phosphorothioate backbone, reported elsewhere (Dias and Stein, 2002; Winkler et al., 2010; Liang et al., 2015) prompted further investigation of the global consequences of 2′ *O*-methyl phosphorothioate AO transfection on cell biology, revealing many significant changes. In contrast, the transfection of (charge-neutral) PMOs in the current study did not disrupt subnuclear structures or induce the formation of DBHS-positive nuclear inclusions. This result was reproducible using multiple delivery techniques, including high dose, gymnotic PMO transfection, transfection of PMO annealed to a DNA leash and complexed with liposome reagent (Gebski et al., 2005), as well as electroporation. It is interesting to consider these differences from a chemical perspective and speculate that the nature of the phosphorothioate linkages are the critical factor in initiating the off-target effects. Vickers et al. (2019) measured binding affinities between NONO and gapmer phosphorothioate AOs and determined that the second RNA recognition motif (RRM2)-AO interaction was driven by electrostatic forces, reinforcing the importance of charge to this interaction.

Interestingly, transfection with the two annealed complementary 2′ *O*-methyl phosphorothioate oligonucleotides averts formation of intranuclear inclusions, with improved antisense activity, relative to that of the 2′ *O*-methyl phosphorothioate AO alone. Most importantly, this did not impact cell viability and prevented the 2′ *O*-methyl phosphorothioate AO off-target effects on expression of transcripts evaluated. In the aforementioned context, it is interesting to consider that double stranded AOs, in the form of siRNAs, are broadly used to induce knockdown of target transcripts. We speculate that delivery of the AO as a double stranded duplex delays interaction of the oligomer backbone with nuclear proteins, enhancing their bioavailability for target engagement and antisense activity. Consequently, our findings of reduced off-target binding by duplex oligonucleotides suggest that steric blocking AOs utilizing all-phosphorothioate modified bases could be delivered as duplexes to reduce off-target effects associated with the backbone chemistry. Nagata and others (Asada et al., 2021; Asami et al., 2021; Nagata et al., 2021) designed fully phosphorothioated gapmers annealed to modified RNA and DNA oligonucleotides to form heteroduplexes, conjugated to cholesterol or α-tocopherol at the 5′ end of the RNA strand, and showed delivery to the central nervous system (CNS) after subcutaneous or intravenous administration in mice and rats. In the context of the CNS, the heteroduplex was far more effective in knocking down the target than the single stranded conjugated gapmer (Nagata et al., 2021). Detailed evaluation and comparison of modified oligonucleotides designed to evoke different mechanisms (steric blocking, RNase H1 activation, antagomirs) prepared as duplex and single stranded conjugates will no doubt open new avenues to improve safety and efficacy of nucleic acid drugs.

The nuclear inclusions observed in phosphorothioate transfected cells are associated with altered nuclear architecture, disturbed gene expression and ultimately, apoptosis. While formation of such large, and apparently irreversible structures would be expected to impact on nuclear biology, the likely consequences of aberrant sequestration of paraspeckle and other nuclear proteins on RNA processing and post transcriptional regulation are of prime concern. Paraspeckles are dynamic, RNA-protein nuclear organelles that occur in the interchromatin space in mammalian cells and are now known to contain over 40 different proteins associated with one architectural long non-coding RNA, *NEAT1_2* [for review see (Fox et al., 2018)]. Observed in most cultured cells-other than various stem cells, and cells experiencing stress, paraspeckles regulate gene expression through sequestration of proteins and RNAs (Fox et al., 2018), and are also implicated in microRNA biogenesis (Jiang et al., 2017). In the process of forming paraspeckles, distinct paraspeckle proteins undergo rapid, but normally reversible interactions with *NEAT1_2* RNA, and each other, culminating in liquid-liquid phase separation to produce the mature paraspeckle. The mechanism by which gapmer phosphorothioate AOs induce some paraspeckle proteins to form aberrant structures is beginning to be teased apart and is influenced by protein interactions as well as the 2′ sugar AO modifications (Bailey et al., 2017; Crooke et al., 2017). How other nuclear proteins, besides DBHS, become incorporated into the phosphorothioate AO induced nuclear inclusions, and why TDP-43, a major component of paraspeckles, does not appear in these inclusions in our study, remains elusive.

Wide-field and Super-resolution fluorescent imaging of 2′ *O*-methyl phosphorothioate transfected fibroblasts revealed the paraspeckle components NONO, PSPC1, SFPQ and FUS co-localised with nuclear inclusions in excess of 2,000 nm in length, with FUS decorating the branch-points and termini. The longitudinal striations apparent by TEM show a striking regularity, with approximately 27 nm spacing of striations, and a diameter of approximately 16 nm for individual striations. These striations are thought to be composed of NONO, SFPQ, PSPC1, and likely contain other proteins. Given these proteins contain coiled-coil domains that can form long extended fibres (Lee et al., 2015), it is intriguing to speculate that the striations are defined by the coiled-coil interactions upon binding to the AO. FUS is found capping ‘bundles’ of these structures in super-resolution images and these correspond to intense electron dense termini. The localization of FUS is somewhat reminiscent of FUS low complexity domain localizing at 45 nm intervals along intermediate filaments, formed by vimentin, where FUS interacts with the disordered head domain of vimentin at these sites. Intermediate filament proteins, including vimentin, also employ coiled-coil domains to form filaments (Zhou et al., 2021). Thus, the AO-induced nuclear inclusions could be a synthetic-scaffolded nuclear intermediate filament equivalent that warrants further investigation from a structural and cellular perspective.

Coilin, nucleolin, fibrillarin, and SC35 (SRSF2), all showed altered distribution in cells that have phosphorothioate AO-induced nuclear inclusions, but largely did not co-localize with these inclusions. Cajal bodies assemble spliceosomal and nucleolar ribonucleoproteins required for pre-mRNA and pre-rRNA processing, and are recently proposed to contribute to genome organization, with global effects on gene expression and RNA splicing (Wang et al., 2016). The nucleolus is the most prominent nuclear structure and is where synthesis and processing of ribosomal transcripts to yield the mature rRNAs *5.8S, 18S* and *28S* from the *45S* pre-rRNA takes place. We show that ribosomal RNA processing is greatly impaired by 2′ *O*-methyl phosphorothioate AO transfection; and we speculate that this is a likely manifestation of improper localization of major protein components of the nucleolus and Cajal bodies and consequent functional disruption. Our results reflect the perturbations caused by gapmer phosphorothioate AOs whereby *5.8S* rRNA becomes sequestered in nuclear inclusions, with consequential nucleolar stress (Shen et al., 2019). In addition, the aberrant inclusions that we suggest occupy the inter chromosomal spaces may well impose physical constraints upon nuclear organisation, and prevent proper localisation of Cajal bodies and the nucleoli close to their normal chromosomal sites. In cells with a high demand for transcript splicing and ribosome biogenesis, such as neurons that have prominent Cajal bodies, juxtaposed to nucleoli [for review see (Lafarga et al., 2017)], disruption or loss of Cajal bodies is associated with severe neuronal dysfunction (Tapia et al., 2017). Indeed, disruption or depletion of Cajal bodies was seen as the earliest nuclear sign of motor neuron degeneration in a spinal muscular atrophy mouse model, with a progressive nucleolar dysfunction in ribosome biogenesis (Tapia et al., 2017).

Paraspeckle biology has gained increasing interest due to the association of paraspeckle proteins with neurodegenerative disease, in particular ALS, and the observation that *NEAT1_2* is up-regulated in early-stage motor neurons from the spinal cords of ALS patients (Shelkovnikova et al., 2014; Yamazaki and Hirose, 2015). Paraspeckle formation, not observed in healthy spinal motor neurons, is enhanced in spinal cords of patients with early stage sporadic and familial ALS, and mutations in many paraspeckle proteins (e.g., TDP-43, FUS, NONO, SFPQ) are associated with ALS (Nishimoto et al., 2013; Shelkovnikova et al., 2018). Interestingly, paraspeckles form in a stress granule-dependent manner in some stress contexts (An et al., 2019). This inter-dependence suggests a continuum of RNA-protein granules important for cellular homeostasis, providing further reason to avoid therapeutic agents that perturb these natural structures. Whether paraspeckles are protective or causative in ALS molecular pathology is not known at this time (Yamazaki and Hirose, 2015). Nevertheless, phosphorothioate AO-mediated dysregulation of paraspeckle formation and altered nucleoli and Cajal body biology shown in our study, together with the building body of evidence that nuclear organelle dysfunction (Lafarga et al., 2017; Tapia et al., 2017) has implications for central nervous system, and probably all, clinical applications of these compounds.

Not surprisingly, considering the altered nuclear architecture and distribution of proteins implicated in RNA biology, transcriptome sequencing showed significant global effects on the expression of transcripts and revealed disturbances to many critical cellular processes in phosphorothioate AO transfected cells. Of particular note, pathways involved in apoptosis, chromatin silencing, cellular metabolism and a number of signalling pathways, autophagy and nucleotide excision repair were disturbed. While we acknowledge that our *in vitro* studies in replicating cells may not fully reflect potential off-target treatment effects in tissues *in vivo*, Toonen et al. (2018) report significant upregulation of immune system-associated genes in brains of mice treated by intracerebroventricular injection of 2′ *O*-methyl fully-phosphorothioate AO. The upregulation of immune system associated genes was detectable for at least 2 months after the final AO administration. In our study, the exaggerated sequestration of nuclear proteins resulting from 2′ *O*-methyl phosphorothioate AO transfection at 100 nM dramatically disturbs RNA processing, disrupts nuclear architecture, and induces apoptosis. In this light, it seems reasonable to consider that localized injection site reactions and adverse effects reported after clinical evaluation of the 2′ *O*-methyl fully-phosphorothioated AO *Drisapersen* for the treatment of Duchenne muscular dystrophy, supplied and injected at 28.6 µM (Flanigan et al., 2014), (Voit et al., 2014; Hilhorst et al., 2019) and gapmer phosphorothioate AO *Kynamro* ^*®*^ (*Mipomersen*), injected at 26.3 µM, for the treatment of familial hypercholesterolemia (Wong and Goldberg, 2014) may be mediated at least in part, by non-specific interactions of nuclear components with the phosphorothioate backbone. It might also be prudent to deliberate on the reported effects of phosphorothioate-containing antisense compounds, attributed to the AO action on the target transcript, and consider whether some level of the apparent antisense effect on gene expression could perhaps be a consequence of disturbance of RNA processing pathways more broadly and altered expression of house-keeping genes. *ACTB*, *TUBB*, *GAPDH*, *CCND1* and many other transcripts/proteins routinely used as housekeeping controls were up or down-regulated significantly by phosphorothioate AO transfection, potentially confounding gene expression studies.

Global effects on the transcriptome, and specifically, pre-mRNA splicing adds further concern to the clinical and *in vitro* application of phosphorothioate AOs, particularly for those fully-modified AOs intended to modulate splicing. Changes in splicing factor expression have been linked to numerous diseases, thus, therapeutics with broad unintended effects on splicing factor expression could have serious implications. Importantly, given our observations on transcript expression following phosphorothioate AO transfection, caution is recommended in data interpretation for the *in vitro* evaluation and screening of such compounds. Further, given the recent increased interest in developing AOs to treat CNS disorders and neurodegenerative diseases, observed changes in expression of genes linked to these disorders should be considered when developing AOs for clinical application. Loss of function mutations and haploinsufficiency has been linked to ALS, with a handful of ALS-associated genes notably downregulated following phosphorothioate AO transfection in this study. While our short-term studies were performed in fibroblasts, over time, cumulative small changes in expression of these genes, following repeated clinical exposure to phosphorothioate AOs may be problematic in long-lived cells.

We speculate that, unlike endogenous paraspeckles and other dynamic nuclear bodies, the formation of the aberrant nuclear inclusions seen *in vitro* here does not appear to be reversible. While 2′ *O*-methyl phosphorothioate AO transfected cultures showed reduced cell numbers, whether this was due to cell death or impaired replication, or both, is uncertain, and whether the nuclear inclusions *per se* or reduced availability of RNA processing are primarily responsible requires further investigation. However, any effects resulting in perturbation of nuclear proteins may be compounded by the formation of the nuclear amyloid-like aggregates and likely disturbance of protein homeostasis, termed ‘proteostasis’ (Yerbury et al., 2016). Although other descriptions of exogenously induced nuclear amyloid-like aggregates are limited, Arnhold et al. (2015) identified large amyloid-like aggregates in SH-SY5Y cells, treated with mercury, a notorious neurotoxicant, in a study that explains the mechanism of heavy metal neurotoxicity and identified amyloid protein aggregation in the cell nucleus as causative. Mass spectrometry of the purified protein aggregates identified a subset of spliceosomal components and the nuclear envelope protein lamin B1 (Arnhold et al., 2015). In our study, we also detected changes in lamin B1, although here we did not detect lamin B1 in the nuclear inclusions, we nevertheless observed that nuclear membrane and lamin B1 organization was distorted in fibroblasts containing nuclear inclusions. Interestingly, the multi-lobulated nuclei and lamin B1 staining are reminiscent of cells from Hutchinson-Gilford progeria patients carrying the lamin A 433G>A mutation (E145K) (Taimen et al., 2009).

In summary, we report phosphorothioate backbone-specific effects of modified single stranded oligonucleotides on the distribution and localization of nuclear proteins, appearance of nuclear structures composed in part of a subset of paraspeckle protein components, and sequence-independent effects on nascent RNA processing. While the *in vivo,* longer-term repercussions of exogenous oligonucleotide-induced nuclear protein aggregates that include many paraspeckle components and cause sub-nuclear disorganisation are yet to be determined, our observations suggest that phosphorothioate backbone antisense compounds destined for clinical application would benefit from further scrutiny.

## Data Availability

The datasets presented in this study can be found online at https://www.ncbi.nlm.nih.gov/geo/query/acc.cgi?acc=GSE121713 (accession number: GSE121713).
