# Supplementary material for: Single Stranded Fully Modified-Phosphorothioate Oligonucleotides can Induce Structured Nuclear Inclusions, Alter Nuclear Protein Localization and Disturb the Transcriptome In Vitro
Source: Front Genet. 2022 Apr 6;13:791416. doi: 10.3389/fgene.2022.791416 (PMC9019733; doi:10.3389/fgene.2022.791416)
Supplement: Supplementary file 3 [file DataSheet1.pdf]

Supplementary Figures:

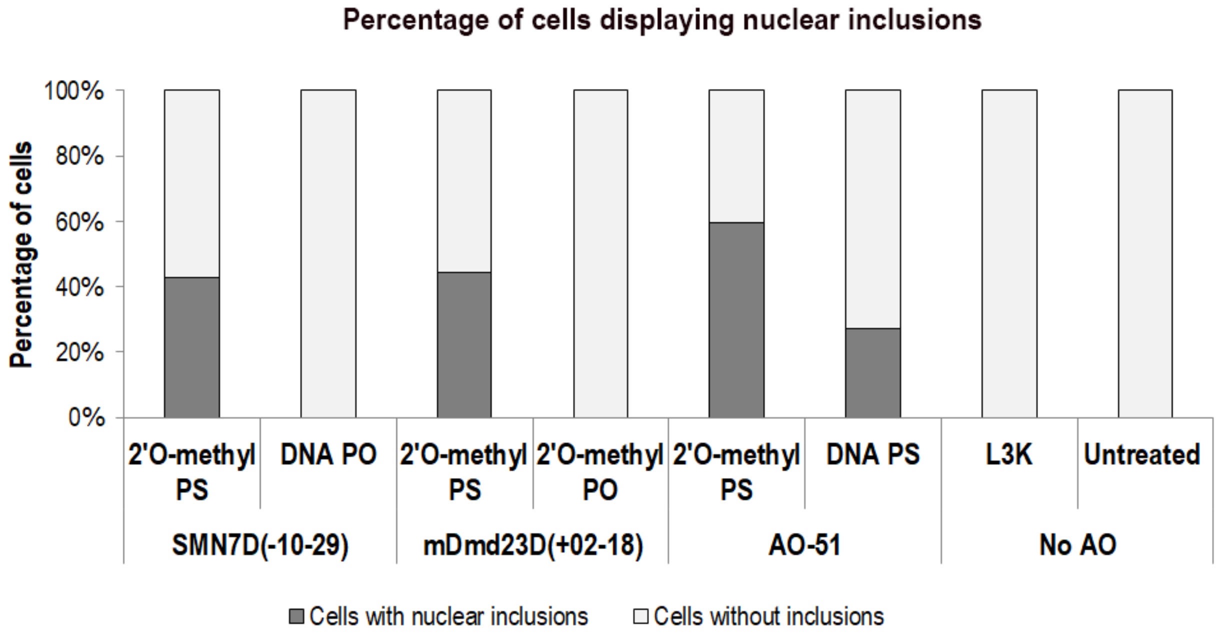

Supplementary Figure S1 – Formation of AO-induced nuclear inclusions associated with phosphorothioate backbone. Graph showing the percentage of fibroblasts containing nuclear inclusions following antisense transfection of different AO chemistries transfected at 100 nM, with a minimum of 200 cells counted per sample. AO chemistries evaluated include: 2´ O-methyl modified bases on a phosphorothioate (PS) backbone (*SMN7D(-10-29)*, *mDmd23D(+02-18*) (Gebski et al. 2005) and AO-51 (**Supplementary Table S3**), DNA unmodified bases on an unmodified PO backbone (*SMN7D(-10-29)*), 2´ O-methyl modified bases on a phosphodiester (PO) backbone (*mDmd23D(+02-18)*), DNA unmodified bases on a PS backbone (AO-51), and compared to fibroblasts treated with Lipofectamine 3000 (L3K) left untreated.

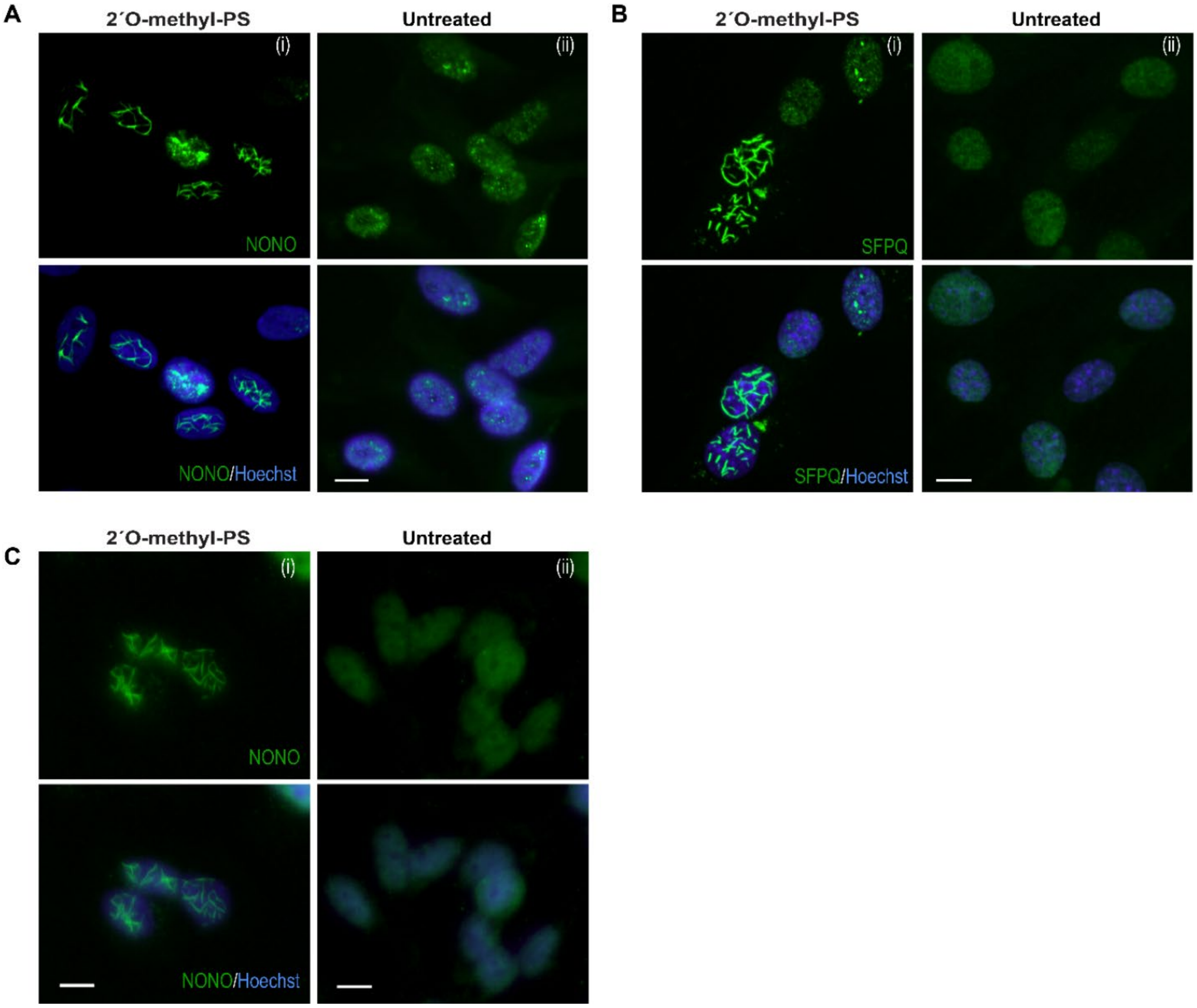

Supplementary Figure S2 – Immunofluorescence staining of paraspeckle proteins in additional cell types. Staining was carried out following transfection with **(A)** (i) 2´ O-methyl phosphorothioate *SMN7D(-10-29)* in primary human myogenic cells; **(B)** (i) 2´ O-methyl phosphorothioate AO targeting *Smn* in mouse myogenic cells; and **(C)** (i) the 2´ O-methyl phosphorothioate control AO in SH-SY5Y neuroblastoma cells. (i) 2´ O-methyl phosphorothioate-transfected and (ii) untreated shown in each panel. NONO was immunostained in human cells and SFPQ in mouse cells. Scale bar = 10 µm.

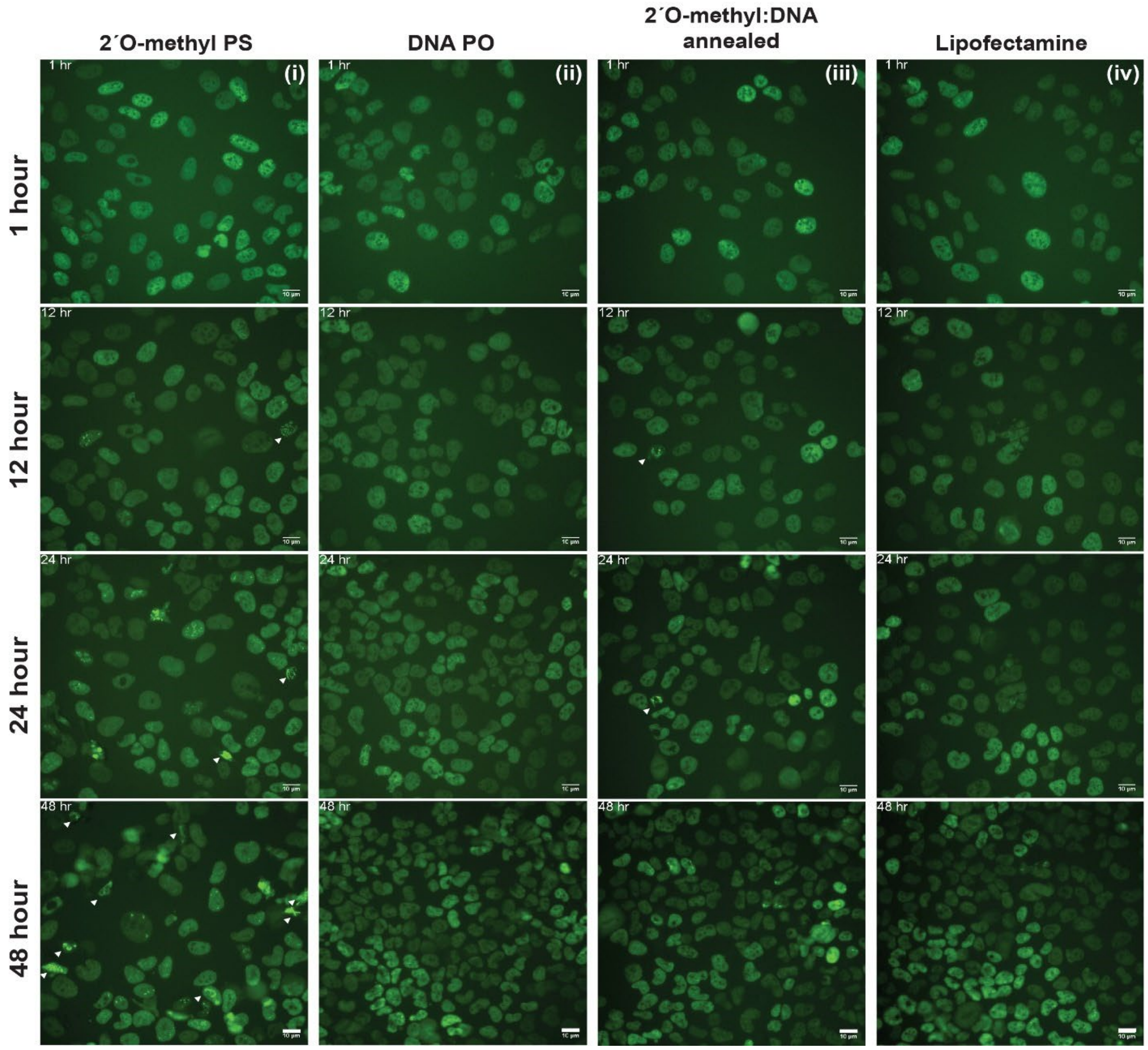

Supplementary Figure S3 – Images from live cell imaging of GFP-SFPQ expressing U2OS cells transfected with 2´ O-methyl phosphorothioate AO and controls. Live cell images were captured following transfection (150 nM) with (i) 2´ O-methyl PS, (ii) DNA PO, (iii) annealed 2´ O-methyl PS and DNA (2´O-methyl:DNA) AOs and (iv) lipofectamine only control over 48 hours, with still images at 1, 12, 24 and 48 hours displayed. Nuclei showing fibril-like SFPQ positive inclusions are indicated with '∆'. Scale bar = 10 μm. Full time-course videos are available in **Supplementary Videos S1-S4**.

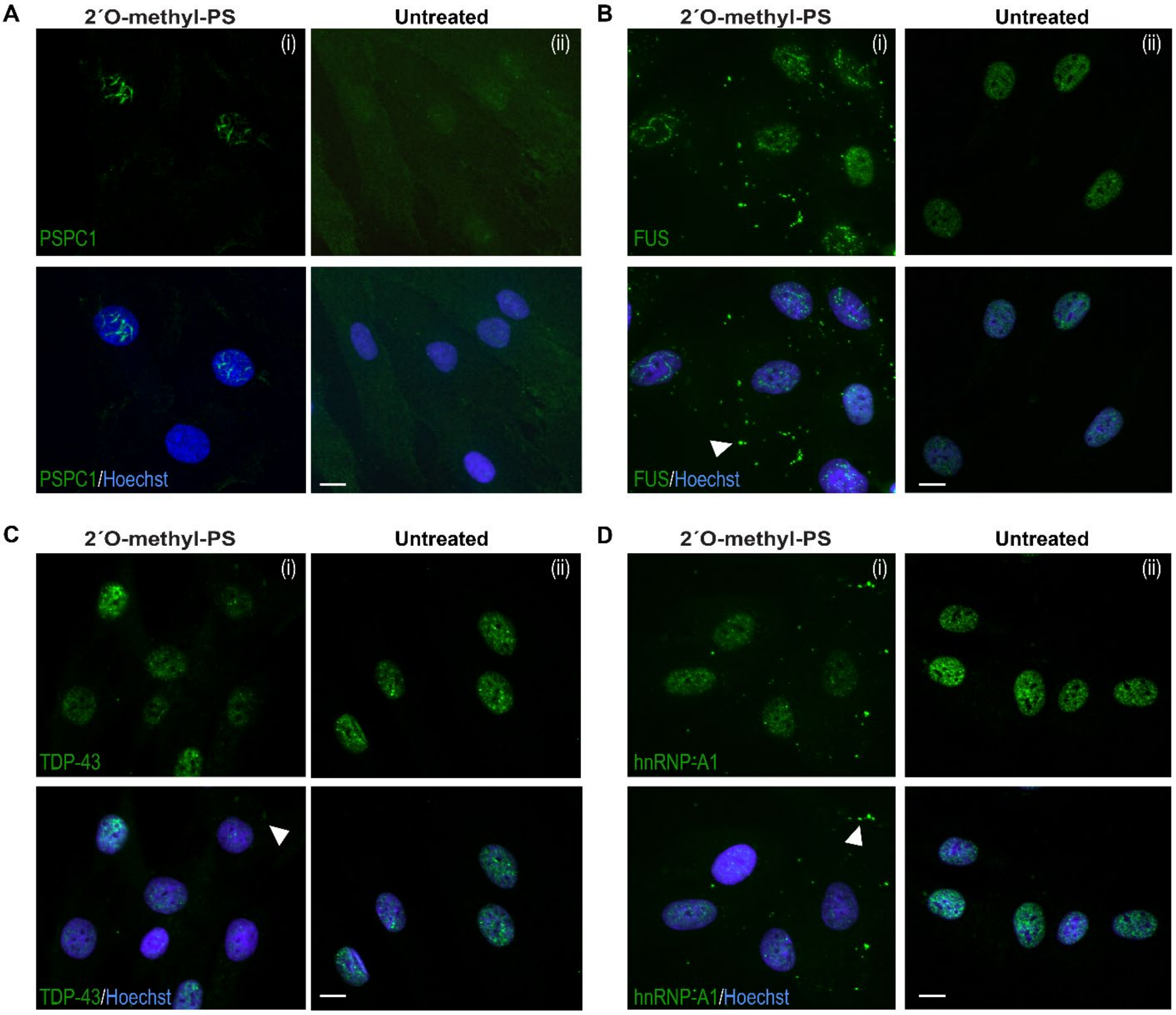

Supplementary Figure S4 - Immunofluorescence staining of additional paraspeckle proteins following 2´ O-methyl phosphorothioate *SMN7D(-10-29)* transfection (100 nM for 24 hours). Showing **(A)** paraspeckle protein component 1 (PSPC1); **(B)** fused in sarcoma (FUS); **(C)** TAR-DNA binding protein 43 (TDP-43); and **(D)** heterogeneous nuclear ribonucleoprotein A1 (hnRNP-A1); in (i) 2´ O-methyl transfected and (ii) untreated cells shown in each panel. The white arrows point to cytoplasmic protein accumulation. Scale bar = 10 µm.

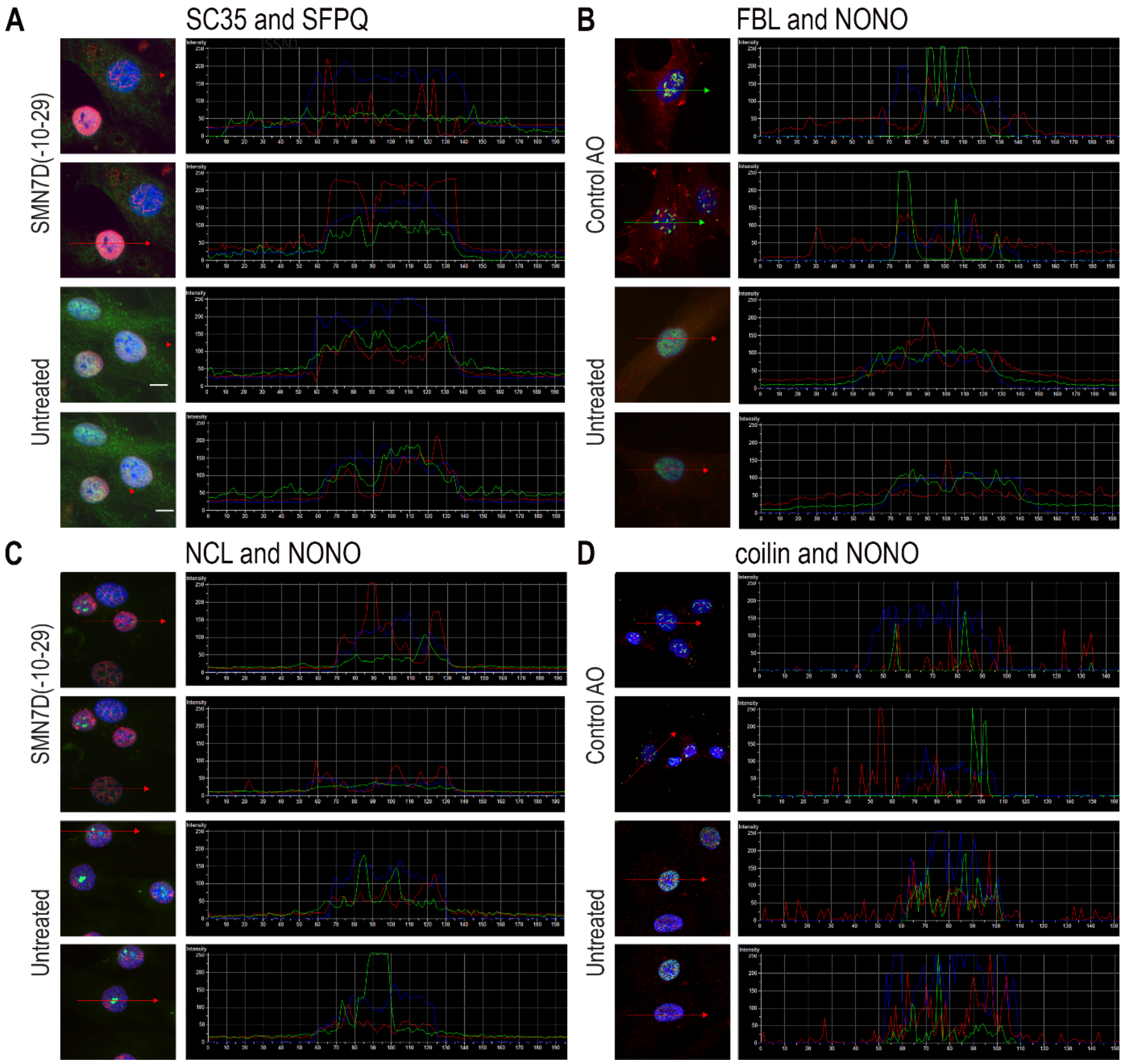

Supplementary Figure S5 – Intensity profiling of immunofluorescent staining of paraspeckle proteins following transfection of the 2′ O- methyl phosphorothioate AOs. Including, *SMN7D(-10-29)* **(A, C)** and the Control AO **(B, D)** (100 nM for 24 hours) and intensity profiling of selected cells showing **(A)** SFPQ (red) and SC35 (green); **(B)** NONO (green) and FBL (red); **(C)** SFPQ (red) and NCL (green); **(D)** NONO (green) and coilin (red).

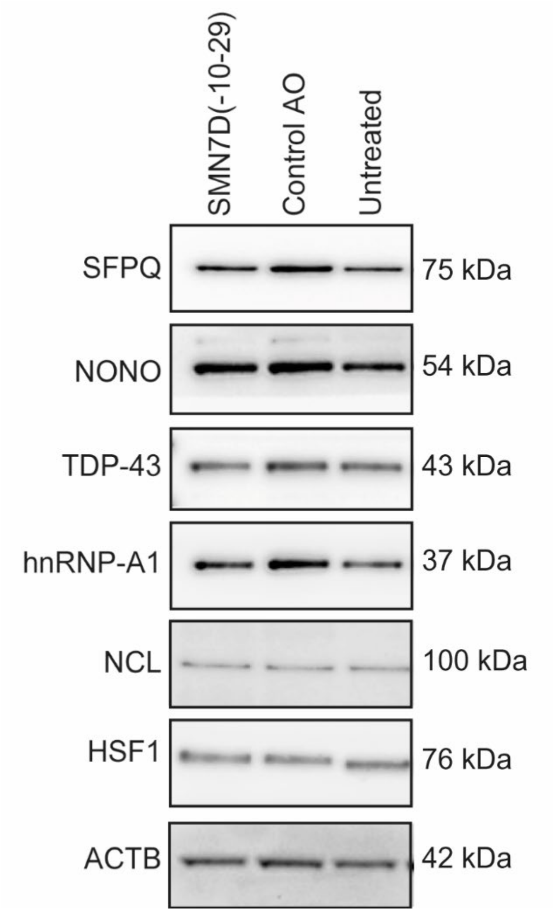

Supplementary Figure S6 – Western blots following 2´ O-methyl phosphorothioate AO transfection. Western blots of extracts from cells transfected with the *SMN7D(-10-29)* and Control AOs (100 nM for 24 hours), probed with antibodies that recognize SFPQ, NONO, TDP-43, hnRNP-A1, NCL, HSF1 and ACTB (**Supplementary Table S2**).

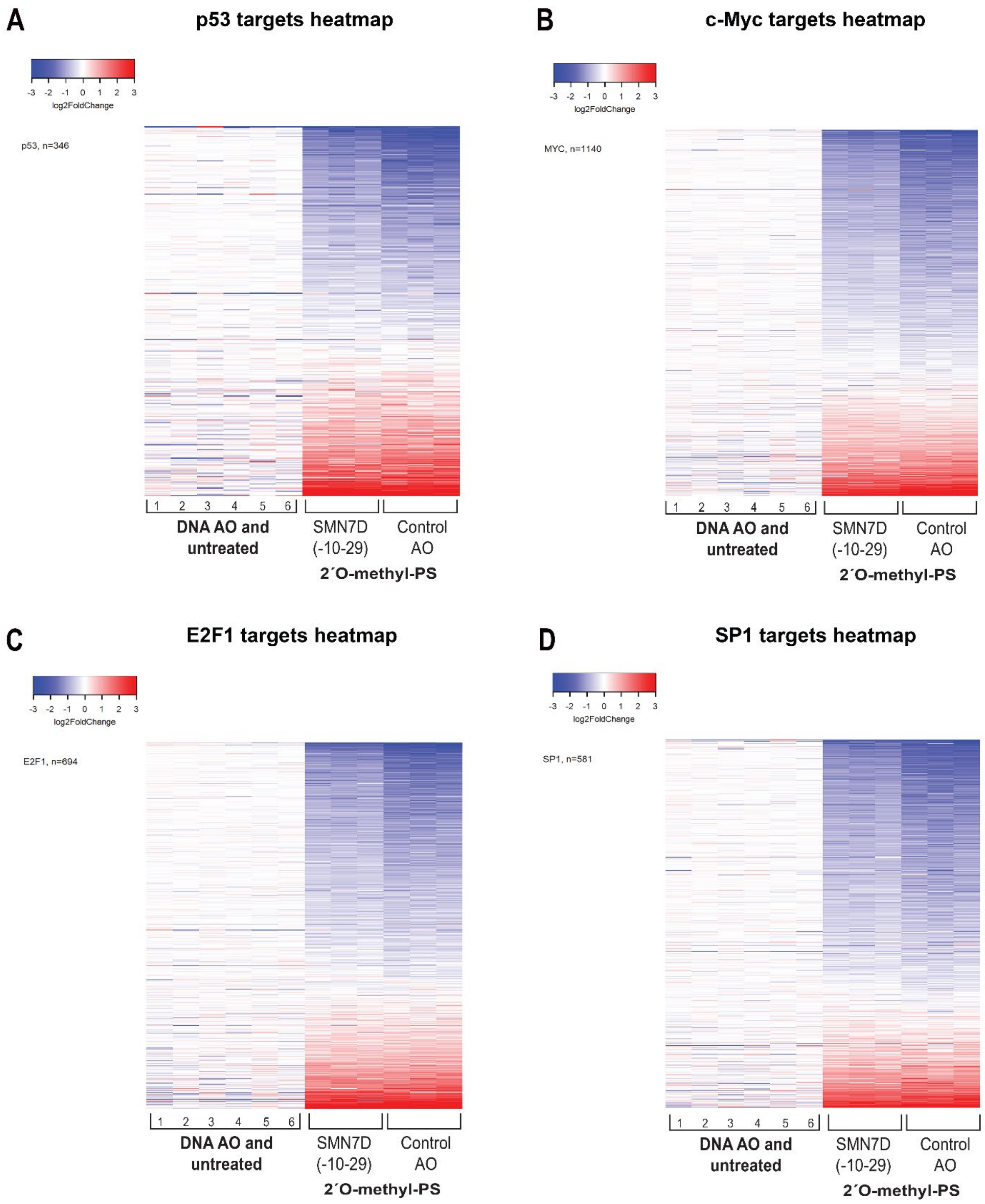

Supplementary Figure S7 – RNAseq heatmaps following 2´ O-methyl phosphorothioate AO transfection. Showing heatmaps of transcripts regulated by the transcription factors involved in nucleolar stress response, p53 **(A)**, c-Myc **(B)**, E2F1 **(C)**, and SP1 **(D)**. The number of transcripts included in each heatmap is indicated.

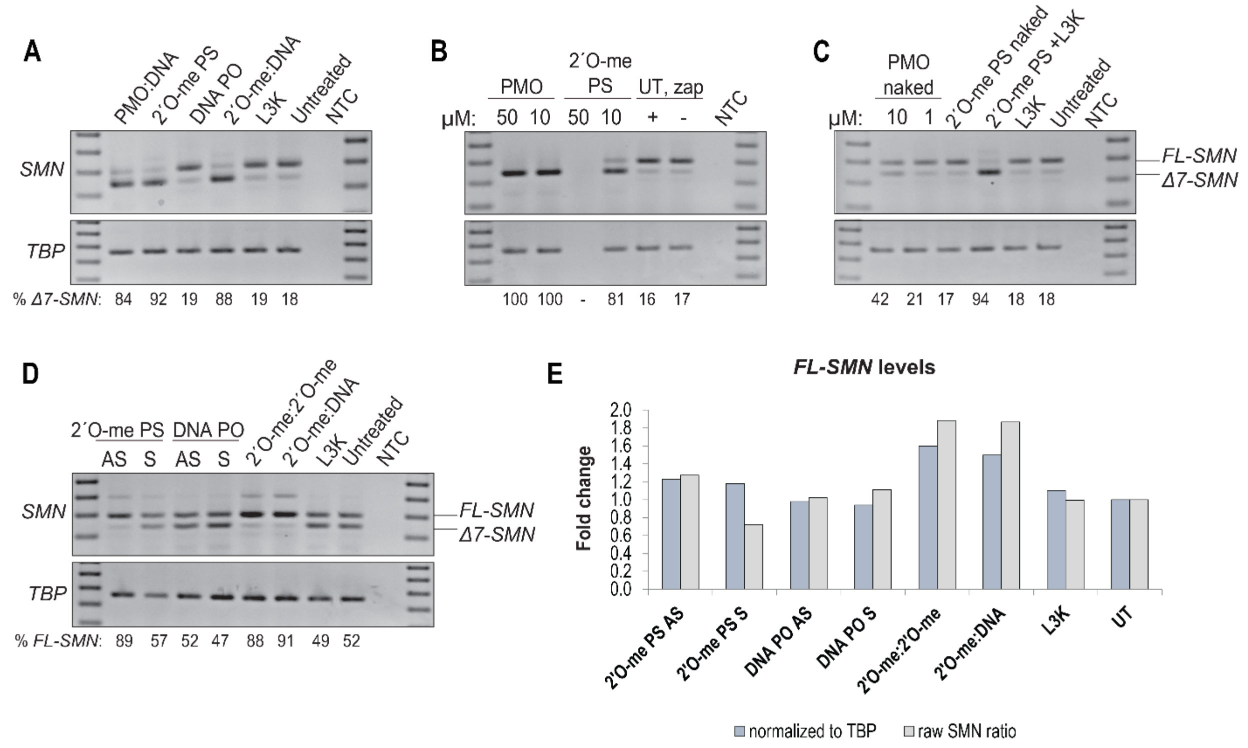

Supplementary Figure S8 – Antisense effect of chemistries that do not induce nuclear inclusions. Representative gel images of RT-PCRs across *SMN* and *TBP* following transfection with the following: **(A)** lipoplex transfection (100 nM) of *SMN7A(+13+32)* sequence as: annealed PMO:DNA leash duplex, 2´ O-methyl phosphorothioate (PS) AO, DNA lease AO sequence (DNA PO) and annealed 2´ O-methyl:DNA duplex using Lipofectamine 3000 (L3K), with the transfection incubated for 24 hours; **(B)** Neon electroporation (50 and 10 μM) of *SMN7A(+13+32)* sequence as PMO and 2´ O-methyl PS chemistries, compared to untreated (UT) cells that underwent the Neon electroporation protocol (zap) and those that did not, and transfection incubated for 24 hours. The 2´ O-methyl PS oligonucleotide ablated transcript expression; **(C)** gymnotic 'naked' oligonucleotide delivery of *SMN7A(+13+32)* sequence as a PMO (10 μM and 1 μM) and 2´ O-methyl PS AO delivered naked (1 μM) and in a lipoplex (L3K, 100 nM) as a positive control for nuclear inclusions, and compared to L3K and untreated control cells, with transfection incubated for 72 hours. There were no viable cells for collection following transfection of the 2´ O-methyl PS sequence at 10 μM for comparison to the PMO sequence; and **(D)** lipoplex transfection of annealed AOs (100 nM), showing the *SMN7D(-10-29)* antisense sequence and complementary sense sequence as 2´ O-methyl PS and DNA PO AOs, annealed 2´ O-me:2´ O-me and 2´ O-me:DNA sequences and compared to the L3K and untreated control cells, with transfection incubated for 24 hours. Normal human fibroblasts were used for **(A-C)**, with the percentage of exon 7 skipped transcripts (% Δ7-*SMN* shown below each lane, and SMA fibroblasts were used for **(D)** with the percentage of full length SMN (% FL-*SMN*) transcripts shown below each lane. A no template control (NTC) was loaded in the final lane and a 100 bp marker used for size comparison; **(E)** graph showing the fold-change in FL-*SMN* levels as in **(D)**, displaying the data normalized to *TBP* and the raw SMN ratio, given that *TBP* expression was affected by transfection with phosphorothioate AOs.

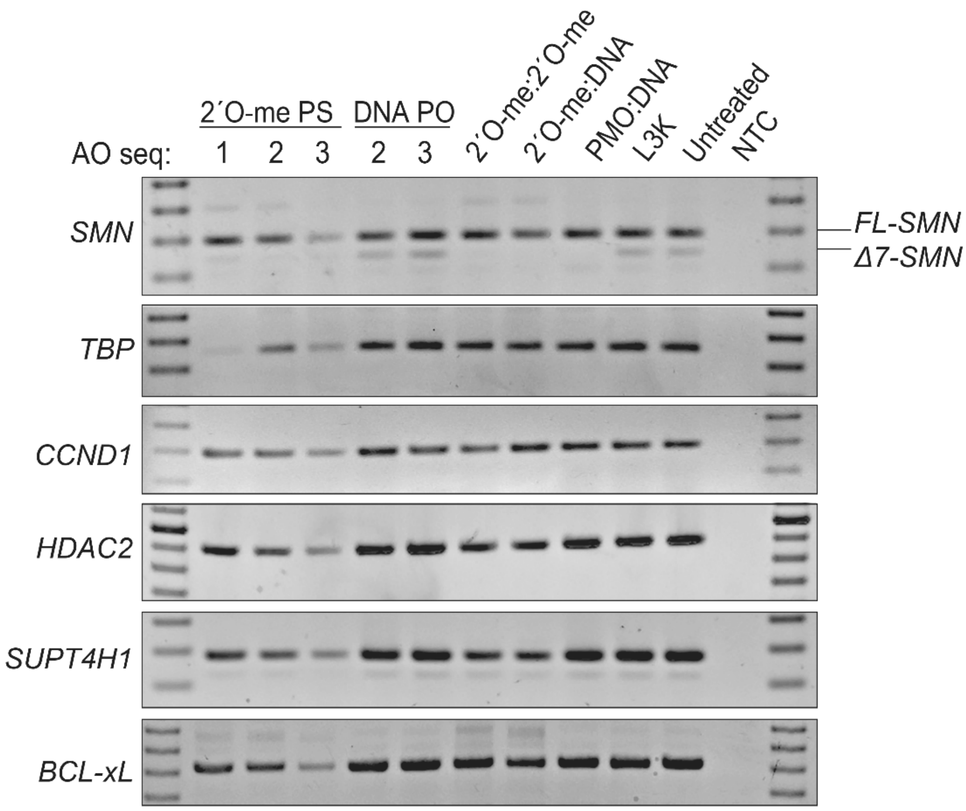

Supplementary Figure S9 – Effect of 2´ O-methyl phosphorothioate (2´O-me PS) AO induced nuclear inclusions on off-target transcript expression following transfection of normal fibroblasts with AO sequences as different chemistries. Representative gel images of RT-PCRs across *SMN* (showing full length (FL) and exon 7 deleted (Δ7) transcripts), *TBP, CCND1, HDAC2, SUPT4H1*, and *BCL-xL* transcripts following transfection with (1) the control sequence AO, (2) *SMN7D(-10-29)* antisense and (3) *SMN7D(-10-29)* sense sequences, synthesized as 2´ O-methyl PS or DNA PO AOs, and as double stranded antisense/sense 2´ O-me:2´ O-me, 2´ O-me:DNA and PMO:DNA AOs, compared to the lipofectamine 3000 (L3K) and untreated transfection controls. A no template control (NTC) was loaded in the final lane and a 100 bp marker used for size comparison.

Supplementary Tables:

Supplementary Table S1 Primer sequences and PCR conditions used in this study.

Primers	Concentration/amount of primer per reaction	Sequence 5´-3´	Temperature profile
SMN	25 ng	AGGTCTCCTGGAAATAAATCAG<br>TGGTGTCATTTAGTGCTGCTCT	55°C 30 min<br>94°C 2 min<br>25 cycles:<br>94°C 40 sec<br>56°C 30 sec<br>68°C 1 min
TBP	25 ng	AGCGCAAGGGTTTCTGGTTT<br>GGAGTCATGGGGGAGGGATA	55°C 30 min<br>94°C 2 min<br>24 cycles:<br>94°C 40 sec<br>565C 30 sec<br>68°C 1 min
BCL2	25 ng	AATGTCTCAGAGCAACCGGG<br>GGGAGGGTAGAGTGGATGGT	55°C 30 min<br>94°C 2 min<br>25 cycles:<br>94°C 40 sec<br>60°C 30 sec<br>68°C 1 min
CCND1	25 ng	TGTTCGTGGCCTCTAAGATGAA<br>GTCCGGGTCACACTTGATCAC	55°C 30 min<br>94°C 2 min<br>23 cycles:<br>94°C 40 sec<br>60°C 30 sec<br>68°C 1 min
HDAC2	25 ng	GCTGTCAATTTTCCAATGAG<br>CAGTCCATGCCAAAGTAGTAT	55°C 30 min<br>94°C 2 min<br>24 cycles:<br>94°C 40 sec<br>60°C 30 sec<br>68°C 1 min
SUPT4H1	25 ng	TTACTTCCTGCGGGTGCACA<br>CTGGATTTGTAGGCCACTCC	55°C 30 min<br>94°C 2 min<br>24 cycles:<br>94°C 40 sec<br>60°C 30 sec<br>68°C 1 min
5S	50 nM	GGCCATACCACCCTGAACGC<br>CAGCACCCGGTATTCCCAGG	95 °C 20 sec<br>40 cycles<br>95 °C 3 sec<br>60 °C 30 sec
18S	250 nM	GTAACCCGTTGAACCCCATT<br>CCATCCAATCGGTAGTAGCG	
45S	100 nM	TGTCAGGCGTTCTCGTCTC<br>AGCACGACGTCACCACATC	
TBP	500 nM	TCAGGCGTTCGGTGGATCGAGT<br>AGTGATGCTGGGCACTGCGGAGAA	
TUBB	100 nM	CTTCGGCCAGATCTTCAGAC<br>AGAGAGTGGGTCAGCTGGAA	

Supplementary Table S2 Antibodies used in this study, indicating dilutions used, detection method and antibody validation.

Protein	Protein name	Supplier	Catalogue #	Dilution IF	Dilution WB	Conjugate	Validation
NONO	Non-POU domain containing octamer	Prepared in house	NA	1:1000	1:10000	mouse	(Souquere, Beauclair et al. 2010)
SFPQ	Splicing factor proline & glutamine rich	Abcam	Ab38148	1:1000	1:8000	rabbit	(Ke, Dramiga et al. 2012)
PSPC1	paraspeckle protein component 1	Merck Millipore	HPA038904	1:50	NA	mouse	Antibodypedia WB, ICC, IHC
FUS	Fused in sarcoma	Santa Cruz	Sc-47711	1:500	NA	mouse	Validated in SW480 cells (https://datasheets.scbt.com/sc-47711.pdf)
TDP-43	TAR-DNA binding protein 43	Protein Tech	10782-2-AP	1:300	1:3000	rabbit	Antibodypedia WB, ICC, IHC
hnRNP - A1	Heterogeneous nuclear ribonucleoprotein A1	Thermo Scientific	PA5-19431	1:700	1:2000	rabbit	Antibodypedia WB, IHC
NCL	Nucleolin	Thermo Scientific	39-6400	1:250	1:2000	mouse	Antibodypedia WB, ICC
FBL	Fibrillarin	Cell Signalling	2639	1:400	NA	rabbit	Antibodypedia WB, ICC
COIL	Coilin	Sapphire Bioscience	GTX112570	1:250	NA	rabbit	Validated in HeLa, HEPG2 and 293T cells, and in cell extracts (http://www.genetex.com/Coilin-antibody-GTX112570.html)
LMNB1	Lamin B1	Protein Tech	12987-1-AP	1:500	NA	rabbit	Antibodypedia WB, ICC, IHC
SC35	Splicing factor SC35	Merk Millipore	04-1550	1:1000	NA	mouse	(Werwein, Dzuganova et al. 2013)

HSF1	Heat shock factor 1	Cell Signaling	12972	1:400	1:500	rabbit	Antibodypedia<br>WB, ICC, IHC
TIA-1	TIA1 cytotoxic granule associated RNA binding protein	Santa Cruz	Sc-48371	1:100	NA	mouse	(Mackenzie, Nicholson et al. 2017)
ACTB	Beta actin	Sigma	A5441	NA	1:60000	mouse	Validated in HS-68(WB), HeLa, JURKAT, COS7, NIH-3T3, PC-12, RAT2, CHO, MDBK, MDCK, FS-11 human fibroblast (ICC)<br>https://www.sigmaaldrich.com/catalog/product/sigma/a5441?lang=en&region=AU
P53	Tumor Protein 53	Novocastra	NCL-Lp53- D07	NA	1:1000	mouse	(Horne, Anderson et al. 1996) (WB)

References:

Horne, G. M., J. J. Anderson, D. G. Tiniakos, G. G. McIntosh, M. D. Thomas, B. Angus, J. A. Henry, T. W. Lennard and C. H. Horne (1996). "p53 protein as a prognostic indicator in breast carcinoma: a comparison of four antibodies for immunohistochemistry." Br J Cancer **73**(1): 29-35.

Ke, Y. D., J. Dramiga, U. Schutz, J. J. Kril, L. M. Ittner, H. Schroder and J. Gotz (2012). "Tau-mediated nuclear depletion and cytoplasmic accumulation of SFPQ in Alzheimer's and Pick's disease." PLoS One **7**(4): e35678.

Mackenzie, I. R., A. M. Nicholson, M. Sarkar, J. Messing, M. D. Purice, C. Pottier, K. Annu, M. Baker, R. B. Perkerson, A. Kurti, B. J. Matchett, T. Mittag, J. Temirov, G. R. Hsiung, C. Krieger, M. E. Murray, M. Kato, J. D. Fryer, L. Petrucelli, L. Zinman, S. Weintraub, M. Mesulam, J. Keith, S. A. Zivkovic, V. Hirsch-Reinshagen, R. P. Roos, S. Zuchner, N. R. Graff-Radford, R. C. Petersen, R. J. Caselli, Z. K. Wszolek, E. Finger, C. Lippa, D. Lacomis, H. Stewart, D. W. Dickson, H. J. Kim, E. Rogaeva, E. Bigio, K. B. Boylan, J. P. Taylor and R. Rademakers (2017). "TIA1 Mutations in Amyotrophic Lateral Sclerosis and Frontotemporal Dementia Promote Phase Separation and Alter Stress Granule Dynamics." Neuron **95**(4): 808-816 e809.

Souquere, S., G. Beauclair, F. Harper, A. Fox and G. Pierron (2010). "Highly ordered spatial organization of the structural long noncoding NEAT1 RNAs within paraspeckle nuclear bodies." Mol Biol Cell **21**(22): 4020-4027.

Werwein, E., M. Dzuganova, C. Usadel and K. H. Klempnauer (2013). "B-Myb switches from Cyclin/Cdk-dependent to Jnk- and p38 kinase-dependent phosphorylation and associates with SC35 bodies after UV stress." Cell Death Dis **4**: e511.

Supplementary Table S3 Results of SFPQ immunostaining following transfection of fibroblasts with 2´ O-methyl phosphorothioate AOs (100 nM, 24 hours). The nucleotide composition and length of each AO is indicated. Cells were stained for SFPQ and the percentages of cells with nuclear inclusions recorded, as was the percentage of cells showing cytoplasmic SFPQ aggregation.

AO #	Nucleotide composition				Length	Cells with nuclear inclusions	Total cell number	SFPQ-nuclear inclusions (% cells)	Cytoplasmic SFPQ staining	Comments
	A	C	G	U						
1	4	9	4	8	25	56	56	**100.0**		
2	5	9	3	8	25	60	60	**100.0**		
3	3	8	3	11	25	107	109	**98.2**		
4	4	10	3	8	25	87	89	**97.8**		
5	4	8	7	6	25	118	121	**97.5**	>90%	28% G content
6	5	11	4	5	25	95	98	**96.9**		
7	6	7	8	4	25	29	30	**96.7**		
8	4	9	3	9	25	112	116	**96.6**		
9	4	7	3	11	25	76	79	**96.2**		
10	7	2	7	9	25	70	73	**95.9**		
11	6	2	3	14	25	128	134	**95.5**		
12	7	8	3	7	25	63	66	**95.5**		
13	7	7	3	8	25	55	58	**94.8**		
14	10	10	5	2	27	78	84	**92.9**		
15	10	7	2	6	25	130	140	**92.9**		
16	4	9	2	9	24	122	132	**92.4**		
17	11	3	9	7	30	101	110	**91.8**		
18	5	10	4	6	25	52	57	**91.2**		
19	4	6	8	7	25	29	32	**90.6**		
20	1	12	0	12	25	33	37	**89.2**		
21	15	2	3	2	22	44	50	**88.0**		
22	11	3	10	2	26	109	124	**87.9**	>90%	38% G content
23	6	6	4	4	20	95	111	**85.6**		
24	7	8	2	8	25	53	63	**84.1**		
25	4	9	2	11	26	118	142	**83.1**		
26	7	8	3	7	25	90	109	**82.6**		
27	5	7	8	6	26	98	119	**82.4**		
28	3	9	8	5	25	98	120	**81.7**		
29	3	14	6	3	26	40	50	**80.0**		
30	3	7	7	8	25	57	72	**79.2**		
31	0	8	3	14	25	88	113	**77.9**		
32	6	9	5	5	25	109	140	**77.9**		
33	18	2	6	3	29	100	129	**77.5**		
34	6	7	5	7	25	62	81	**76.5**		
35	3	10	3	12	28	104	136	**76.5**		
36	14	5	5	4	28	26	35	**74.3**		

AO #	Nucleotide composition				Length	Cells with nuclear inclusions	Total cell number	SFPQ-nuclear inclusions (% cells)	Cytoplasmic SFPQ staining	Comments
	A	C	G	U						
37	3	9	6	7	25	56	77	**72.7**		
38	10	7	4	4	25	52	72	**72.2**		
39	9	8	4	4	25	114	159	**71.7**	>90%	16% G content
40	5	7	5	8	25	77	108	**71.3**		
41	1	9	4	4	18	72	109	**66.1**		
42	8	4	9	4	25	83	126	**65.9**	>90%	36% G content
43	3	10	8	4	25	86	131	**65.6**	>90%	32% G content
44	9	3	10	3	25	75	115	**65.2**	>90%	40% G content
45	8	4	7	6	25	47	74	**63.5**		
46	6	7	2	10	25	100	158	**63.3**		
47	10	3	5	7	25	65	103	**63.1**		
48	7	6	10	2	25	72	115	**62.6**	>90%	40% G content
49	7	7	8	11	33	25	40	**62.5**		
50	6	4	8	2	20	83	137	**60.6**	>90%	40% G content
51	6	6	2	5	19	76	128	**59.4**		
52	4	8	6	7	25	54	93	**58.1**		
53	1	10	11	8	30	36	62	**58.1**		
54	2	7	8	8	25	78	138	**56.5**		
55	6	8	6	5	25	55	100	**55.0**		
56	7	5	8	4	24	68	125	**54.4**		
57	8	4	7	6	25	60	112	**53.6**		
58	5	7	6	7	25	40	77	**51.9**		
59	6	9	6	5	26	95	187	**50.8**		
60	8	4	5	8	25	34	67	**50.7**		
61	6	5	6	8	25	99	207	**47.8**		
62	6	3	13	3	25	23	49	**46.9**	>90%	52% G content
63	9	4	9	3	25	41	89	**46.1**	>90%	36% G content
64	8	7	7	3	25	55	121	**45.5**		
65	6	1	6	11	24	65	146	**44.5**		
66	3	7	8	7	25	57	134	**42.5**		
67	11	9	3	8	31	44	104	**42.3**		
68	3	10	7	5	25	22	55	**40.0**		
69	6	7	9	3	25	48	122	**39.3**	>90%	36% G content
70	2	9	13	1	25	43	114	**37.7**	>90%	52% G content
71	2	7	6	4	19	45	123	**36.6**		
72	6	7	9	3	25	36	104	**34.6**		
73	5	6	3	0	14	45	132	**34.1**		
74	9	7	5	4	25	30	90	**33.3**		
75	6	3	7	9	25	29	88	**33.0**	>90%	36% G content
76	5	10	8	2	25	52	160	**32.5**		
77	5	6	4	5	20	17	59	**28.8**		
78	6	6	8	1	21	33	115	**28.7**		

**AO #**	**Nucleotide composition**				**Length**	**Cells with nuclear inclusions**	**Total cell number**	**SFPQ-nuclear inclusions (% cells)**	**Cytoplasmic SFPQ staining**	**Comments**
	**A**	**C**	**G**	**U**						
79	5	6	7	7	25	33	117	**28.2**		
80	3	8	5	9	25	39	143	**27.3**		
81	3	5	12	5	25	26	96	**27.1**	>90%	48% G content
82	7	8	7	3	25	21	78	**26.9**		
83	6	4	9	6	25	32	122	**26.2**	>90%	36% G content
84	4	10	6	5	25	40	156	**25.6**		
85	4	5	9	5	23	15	134	**11.2**	>90%	39% G content
86	4	3	8	4	19	10	138	**7.2**	>90%	42% G content
87	2	4	11	8	25	8	146	**5.5**	>90%	44% G content
88	6	6	12	3	27	3	169	**1.8**	>90%	44% G content
89	9	4	11	1	25	2	161	**1.2**	>90%	44% G content
90	3	4	12	6	25	0	85	**0.0**		25% of cells show SFPQ localised to nuclear envelope
UT 1						0	122	**0.0**		
UT 2						0	85	**0.0**		

Supplementary Table S4 GO Terms for Genes showing altered expression in 2’ O-methyl phosphorothioate antisense oligonucleotide transfected cells

UPREGULATED GENE EXPRESSION

**NEGATIVE REGULATION OF CAMP SIGNALLING**	**size**	**NES**	**pvalue**	**FDR**
GO_ADENYLATE_CYCLASE_INHIBITING_G_PROTEIN_COUPLED_RECEPTOR_SIGNALING_PATHWAY	67	1.4033092	0.001002004	0.09743188
GO_CAMP_METABOLIC_PROCESS	34	1.4328331	0.011077543	0.0764675
GO_NEGATIVE_REGULATION_OF_CYCLIC_NUCLEOTIDE_METABOLIC_PROCESS	42	1.4055219	0.01002004	0.09573191
GO_ACTIVATION_OF_PROTEIN_KINASE_A_ACTIVITY	17	-1.6124529	0	0.026522638
**STAT3 CASCADE**	**size**	**NES**	**pvalue**	**FDR**
GO_REGULATION_OF_TYROSINE_PHOSPHORYLATION_OF_STAT3_PROTEIN	44	1.4635448	0.004024145	0.057533268
GO_POSITIVE_REGULATION_OF_TYROSINE_PHOSPHORYLATION_OF_STAT3_PROTEIN	37	1.5350006	0	0.02592797
GO_POSITIVE_REGULATION_OF_STAT_CASCADE	72	1.4300276	0.002	0.078029625
GO_REGULATION_OF_TYROSINE_PHOSPHORYLATION_OF_STAT1_PROTEIN	16	1.4674374	0.026041666	0.05570127
**CHROMATIN SILENCING**	**size**	**NES**	**pvalue**	**FDR**
GO_PROTEIN_HETEROTETRAMERIZATION	37	1.5180206	0.001004016	3.24E-02
GO_NEGATIVE_REGULATION_OF_MEGAKARYOCYTE_DIFFERENTIATION	18	1.4655974	0.018480493	0.05672158
GO_NEGATIVE_REGULATION_OF_HEMATOPOIETIC_PROGENITOR_CELL_DIFFERENTIATION	24	1.4251298	0.02123357	0.08160915
GO_CHROMATIN_SILENCING	93	1.5536975	0	0.020343194
GO_CHROMATIN_SILENCING_AT_RDNA	37	1.6677153	0	0.002782914
GO_NEGATIVE_REGULATION_OF_GENE_EXPRESSION_EPIGENETIC	110	1.5046362	0	0.0375754
GO_CENTROMERE_COMPLEX_ASSEMBLY	43	1.4221992	0.002004008	0.08319024

GO_CHROMATIN_ASSEMBLY_OR_DISASSEMBLY	168	1.4074483	0	0.09356921
GO_DNA_PACKAGING	184	1.4209635	0	0.08399753
GO_DNA_REPLICATION_DEPENDENT_NUCLEOSOME_ORGANIZATION	32	1.6551554	0	0.003492132

DOWNREGULATED GENE EXPRESSION

**MEMBRANE AND ORGANELLE ORGANISATION**	**size**	**NES**	**pvalue**	**FDR**
GO_MULTIVESICULAR_BODY_ORGANIZATION	30	-2.6878452	0	0
GO_REGULATION_OF_EXOSOMAL_SECRETION	17	-1.4602777	0.030303031	0.054562636
GO_MULTI_ORGANISM_MEMBRANE_ORGANIZATION	29	-2.1695724	0	0.00119111
GO_CELL_SEPARATION_AFTER_CYTOKINESIS	17	-1.704032	0	0.018966021
GO_MEMBRANE_BUDDING	110	-2.8605185	0	0
GO_VIRION_ASSEMBLY	36	-2.6067855	0	0
GO_UBIQUITIN_DEPENDENT_PROTEIN_CATABOLIC_PROCESS_VIA_THE_MULTIVESICULAR_BODY_SORTING_PATHWAY	16	-1.8037659	0	0.01173263
GO_MULTI_ORGANISM_ORGANELLE_ORGANIZATION	23	-2.6754305	0	0
**NEGATIVE REGULATION OF AUTOPHAGY**	**size**	**NES**	**pvalue**	**FDR**
GO_NEGATIVE_REGULATION_OF_AUTOPHAGY	51	-1.8540518	0	0.009771321
GO_NEGATIVE_REGULATION_OF_ORGANELLE_ASSEMBLY	22	-1.4039384	0	0.0703511
GO_REGULATION_OF_AUTOPHAGOSOME_ASSEMBLY	34	-2.414632	0	2.78E-04
GO_NEGATIVE_REGULATION_OF_RESPONSE_TO_EXTRACELLULAR_STIMULUS	33	-1.4719748	0	0.051657468
GO_REGULATION_OF_VACUOLE_ORGANIZATION	40	-1.9451795	0	0.006435364
GO_NEGATIVE_REGULATION_OF_MACROAUTOPHAGY	20	-2.5849924	0	0
**NUCLEOTIDE EXCISION REPAIR**	**size**	**NES**	**pvalue**	**FDR**
GO_GLOBAL_GENOME_NUCLEOTIDE_EXCISION_REPAIR	32	-2.953256	0	0
GO_NUCLEOTIDE_EXCISION_REPAIR_DNA_DAMAGE_RECOGNITION	23	-3.7156785	0	0

GO_NUCLEOTIDE_EXCISION_REPAIR_DNA_INCISION	39	-2.1080408	0	0.002141136
GO_NUCLEOTIDE_EXCISION_REPAIR_DNA_DUPLEX_UNWINDING	22	-3.3069005	0	0
GO_NUCLEOTIDE_EXCISION_REPAIR_PREINCISION_COMPLEX_ASSEMBLY	29	-2.947477	0	0
GO_NUCLEOTIDE_EXCISION_REPAIR_PREINCISION_COMPLEX_STABILIZATION	21	-3.1554484	0	0
**PROTEIN TRANSMEMBRANE TRANSPORT**	**size**	**NES**	**pvalue**	**FDR**
GO_MITOCHONDRIAL_TRANSMEMBRANE_TRANSPORT	37	-1.6415616	0	0.023881914
GO_PEROXISOME_ORGANIZATION	33	-3.12492	0	0
GO_PROTEIN_TRANSMEMBRANE_TRANSPORT	49	-1.6217597	0	0.025787786
GO_PEROXISOMAL_TRANSPORT	18	-2.0793462	0	0.002849037
GO_PROTEIN_TARGETING_TO_MITOCHONDRION	46	-1.5986532	0	0.028202297
**NEGATIVE REGULATION OF INTRINSIC APOPTOSIS**	**size**	**NES**	**pvalue**	**FDR**
GO_NEGATIVE_REGULATION_OF_ENDOPLASMIC_RETICULUM_STRESS_INDUCED_INTRINSIC_APOPTOTIC_SIGNALING_PATHWAY	17	-2.4685698	0	0
GO_PROTEIN_EXIT_FROM_ENDOPLASMIC_RETICULUM	18	-3.2342908	0	0
GO_REGULATION_OF_ENDOPLASMIC_RETICULUM_STRESS_INDUCED_INTRINSIC_APOPTOTIC_SIGNALING_PATHWAY	28	-1.5667129	0	0.03213714
GO_REGULATION_OF_ERAD_PATHWAY	27	-2.0681918	0	0.003088701
GO_ENDOPLASMIC_RETICULUM_TO_CYTOSOL_TRANSPORT	21	-1.874271	0	0.008970704
GO_ER_ASSOCIATED_UBIQUITIN_DEPENDENT_PROTEIN_CATABOLIC_PROCESS	58	-3.2112198	0	0
GO_NEGATIVE_REGULATION_OF_RESPONSE_TO_ENDOPLASMIC_RETICULUM_STRESS	35	-2.1436315	0	0.001386112
GO_REGULATION_OF_PROTEIN_EXIT_FROM_ENDOPLASMIC_RETICULUM	18	-1.8251425	0	0.010902868

Supplementary Table S5 Select genes that are differentially expressed following transfection with phosphorothioate AOs, *p*-adj<0.05

Gene Function	***Gene***	***baseMean***	***log2FoldChange***	***lfcSE***	***stat***	***pvalue***	***padj***
Paraspeckle formation	*NONO*	6814.6533	-1.605590056	0.064551	-24.8734	1.45E-136	1.33E-134
	SFPQ	6086.05841	-0.245044029	0.037328	-6.56456	5.22E-11	2.52E-10
	PSPC1	933.5484	0.52539659	0.115657	4.54272	5.55E-06	1.73E-05
	HNRNPA1	23097.6534	-0.875714943	0.043203	-20.2697	2.38E-91	1.02E-89
Long non-coding RNAs	*NEAT1*	28289.7185	0.10944579	0.04473033	2.44679119	0.01441343	0.02555364
	MALAT1	13988.0571	1.36100169	0.08989247	15.1403301	8.78E-52	1.68E-50
Housekeeping genes	*ACTB*	17858.7484	-1.644129341	0.070272	-23.3968	4.61E-121	3.34E-119
	GAPDH	75225.3041	-1.508429767	0.06004	-25.1237	2.74E-139	2.67E-137
	LMNA	35516.4004	-1.190850219	0.118312	-10.0653	7.86E-24	6.77E-23
	TUBB	26558.6187	-1.504779123	0.054218	-27.7543	1.55E-169	2.59E-167
	CCND1	7899.31335	1.501003895	0.147043	10.20795	1.83E-24	1.62E-23
ALS/neurodegenerative disease linked	*TARDBP*	3241.25009	-1.085496089	0.123341	-8.80079	1.36E-18	9.65E-18
	SOD1	3081.30977	-1.635554126	0.054046	-30.262	3.62E-201	1.02E-198
	SQSTM1	26827.0606	-1.014363155	0.08889	-11.4115	3.67E-30	3.88E-29
	NEK1	1221.97681	-1.023991002	0.111042	-9.22166	2.93E-20	2.22E-19
	SFPQ	6086.05841	-0.245044029	0.037328	-6.56456	5.22E-11	2.52E-10
	HNRNPA1	23097.6534	-0.875714943	0.043203	-20.2697	2.38E-91	1.02E-89
	VCP	8459.1865	-1.131364753	0.06775	-16.6991	1.33E-62	3.23E-61
Regulation of autophagy	*BECN1*	1813.02266	-0.975523569	0.06613	-14.7517	3.00E-49	5.39E-48
	LAMP2	6554.77054	-2.07196222	0.080585	-25.7115	8.69E-146	9.59E-144
	MAP1LC3B2	27.3430712	1.580493242	0.654944	2.413173	0.015814298	0.027771015
	ATG4A	388.318602	-0.963138891	0.097783	-9.8498	6.87E-23	5.74E-22
	ATG4B	1409.18523	-1.081389304	0.113684	-9.51223	1.87E-21	1.48E-20
	ATG4C	301.842951	-2.053143566	0.166816	-12.3078	8.22E-35	1.01E-33
Mitochondrial genes	*PINK1*	1938.70279	-1.622947881	0.064272	-25.2514	1.09E-140	1.08E-138
	OMA1	447.345587	-1.036980543	0.122901	-8.4375	3.24E-17	2.18E-16
	TOMM7	1922.14858	-1.583279452	0.092241	-17.1646	4.89E-66	1.27E-64
	TOMM20	3854.23598	-0.988688218	0.043732	-22.6079	3.62E-113	2.33E-111
	OPA1	1447.40171	-0.90932172	0.069156	-13.1488	1.73E-39	2.42E-38

Gene Function	*Gene*	baseMean	log2FoldChange	lfcSE	stat	pvalue	padj
	PRDX3	2235.39048	-1.55696596	0.089438	-17.4083	7.14E-68	1.93E-66
	NDUFS1	2307.52968	-0.783666494	0.055681	-14.0743	5.47E-45	8.82E-44
Chromatin regulators	*CHD2*	9845.30805	1.395002402	0.10683	13.05814	5.71E-39	7.88E-38
	CHD3	4659.65126	-0.972508898	0.171684	-5.66454	1.47E-08	5.90E-08
	CHD4	73.2413445	-0.924334767	0.266846	-3.46393	0.000532354	0.001256052
	CHD5	110.708368	1.668235463	0.295975	5.6364	1.74E-08	6.91E-08
	CHD6	2140.10813	-0.836570193	0.098323	-8.50836	1.76E-17	1.20E-16
	CHD7	244.587533	0.778307314	0.187961	4.140802	3.46E-05	9.75E-05
	BRD7	1423.56572	-0.755449285	0.080551	-9.37855	6.69E-21	5.20E-20
	RSF1	1548.89327	-0.739313131	0.116281	-6.35801	2.04E-10	9.48E-10
	PRMT2	2829.44457	-1.851853336	0.06107	-30.3232	5.67E-202	1.63E-199
	ATM	3402.86124	-0.909411474	0.152626	-5.95842	2.55E-09	1.09E-08
SRSF splicing factors	*SRSF5*	4001.59255	-0.850527896	0.061477	-13.835	1.57E-43	2.44E-42
	SRSF7	2358.80632	1.14924152	0.088445	12.99382	1.33E-38	1.81E-37
	SRSF9	1806.30131	-1.178033031	0.098895	-11.9119	1.03E-32	1.19E-31
	SRSF11	4883.40282	-1.015711402	0.060073	-16.908	3.93E-64	9.77E-63
	SRSF12	28.2567659	1.990821281	0.441186	4.512433	6.41E-06	1.98E-05
hnRNP splicing factors	*HNRNPA0*	2403.48685	-1.205722935	0.051636	-23.3502	1.37E-120	9.78E-119
	HNRNPA1	23097.6534	-0.875714943	0.043203	-20.2697	2.38E-91	1.02E-89
	HNRNPA3	6190.9897	-0.809492026	0.049872	-16.2312	3.03E-59	6.86E-58
	HNRNPC	9339.52659	-0.909265261	0.09021	-10.0794	6.81E-24	5.88E-23
	HNRNPD	4261.0317	-0.707086349	0.071658	-9.86747	5.76E-23	4.83E-22
	HNRNPH1	9072.23317	-0.818582115	0.06308	-12.9769	1.65E-38	2.25E-37
	HNRNPH2	1920.93719	-1.309220659	0.068468	-19.1215	1.67E-81	5.98E-80
	HNRNPK	14247.3337	-0.904650207	0.047333	-19.1124	1.99E-81	7.10E-80
	HNRNPM	3978.32117	-1.017471725	0.069193	-14.7048	6.01E-49	1.08E-47
	HNRNPU	9355.26679	-0.729289831	0.052105	-13.9965	1.64E-44	2.62E-43
PRPF splicing factors	*PRPF4B*	2099.39047	-0.734130085	0.085553	-8.58096	9.41E-18	6.46E-17
	PRPF6	2011.10598	-1.414788414	0.067575	-20.9366	2.48E-97	1.20E-95
	PRPF8	8289.29802	-0.778149686	0.092561	-8.40686	4.21E-17	2.81E-16
	PRPF19	1779.67526	-1.08301036	0.17801	-6.08398	1.17E-09	5.15E-09

Gene Function	*Gene*	baseMean	log2FoldChange	lfcSE	stat	pvalue	padj
	PRPF31	1199.46733	-0.796257916	0.141759	-5.61699	1.94E-08	7.70E-08
	PRPF38A	852.021328	-0.917161319	0.08627	-10.6313	2.13E-26	2.00E-25
	PRPF40A	2777.59937	-0.725634522	0.12483	-5.81296	6.14E-09	2.54E-08
	PRPF40B	513.828913	-0.917683411	0.135945	-6.75039	1.47E-11	7.38E-11
HDAC family	*HDAC1*	1837.61759	-1.440049011	0.07919	-18.1847	6.83E-74	2.10E-72
	HDAC10	1006.19949	-0.827671508	0.12377	-6.68715	2.28E-11	1.13E-10
	HDAC11	436.75663	-1.635530783	0.35582	-4.59651	4.30E-06	1.35E-05
	HDAC2	2064.6272	-0.928257864	0.078687	-11.7969	4.05E-32	4.58E-31
	HDAC5	2943.7166	-2.473781357	0.136808	-18.0822	4.40E-73	1.33E-71
	HDAC6	769.893359	-1.744486804	0.10855	-16.0707	4.09E-58	9.03E-57
	HDAC7	4326.56583	-1.094712095	0.076709	-14.2709	3.32E-46	5.52E-45
	HDAC8	360.473621	-1.221989305	0.192027	-6.36365	1.97E-10	9.15E-10
	HDAC9	1741.43601	1.635384808	0.084663	19.31631	3.92E-83	1.45E-81
Transcription factors	*MYC*	2687.38979	2.035542877	0.083492	24.38001	2.79E-131	2.41E-129
	SP1	2836.01523	-1.660449115	0.107005	-15.5175	2.64E-54	5.39E-53
	TP53	1636.18803	-0.825524365	0.173279	-4.76414	1.90E-06	6.23E-06
	E2F1	118.347932	1.414721068	0.261772	5.404393	6.50E-08	2.47E-07
	SUPT3H	240.185055	-1.060643369	0.144788	-7.32549	2.38E-13	1.32E-12
	SUPT4H1	1093.53484	-1.33995859	0.107766	-12.4339	1.71E-35	2.14E-34
	SUPT5H	3813.92856	-0.956058556	0.086227	-11.0877	1.44E-28	1.45E-27
	SUPT7L	1668.44434	-0.79980986	0.069278	-11.545	7.83E-31	8.47E-30
	SUPT20HL1	3.30043215	4.82121153	1.736145	2.776963	0.005486943	0.010684753
	SUPT20HL2	7.54434931	1.996950871	0.712557	2.802513	0.005070619	0.009942881
Stress markers	*NPM1*	16411.8445	-1.41075702	0.07375	-19.1289	1.45E-81	5.20E-80
	FBL	1552.17687	-1.039356066	0.075219	-13.8178	1.99E-43	3.09E-42
	TIA1	1780.67043	-1.021998961	0.110259	-9.26909	1.88E-20	1.43E-19
	G3BP1	3763.72243	-0.715425201	0.082395	-8.68287	3.86E-18	2.69E-17
	NOP2	1120.71496	0.927145476	0.105057	8.82519	1.09E-18	7.79E-18

Supplementary Videos Figure Legend

Supplementary Videos S1-S4 – U2OS cells expressing GFP tagged SFPQ were transfected with various antisense oligomers and imaged for 48 hours immediately after transfection. Including: **Video S1:** transfection with 2'O-methyl PS antisense oligomer alone; **Video S2:** transfection with DNA PO antisense oligomer; **Video S3:** transfection with 2'O-methyl PS antisense oligomer annealed with sense DNA PO oligomer; **Video S4:** cells treated with lipofectamine transfection reagent alone.
